# Poly(ADP-ribose) polymerase 9 mediates early protection against *Mycobacterium tuberculosis* infection by regulating type I IFN production

**DOI:** 10.1172/JCI158630

**Published:** 2023-06-15

**Authors:** Shyamala Thirunavukkarasu, Mushtaq Ahmed, Bruce A. Rosa, Mark Boothby, Sung Hoon Cho, Javier Rangel-Moreno, Stanley K. Mbandi, Valérie Schreiber, Ananya Gupta, Joaquin Zuniga, Makedonka Mitreva, Deepak Kaushal, Thomas J. Scriba, Shabaana A. Khader

**Affiliations:** 1 Department of Molecular Microbiology,; 2 McDonnell Genome Institute, and; 3 Division of Infectious Diseases, Department of Internal Medicine, Washington University in St. Louis, St. Louis, Missouri, USA.; 4 Department of Pathology, Microbiology, and Immunology, Vanderbilt University Medical Center, Nashville, Tennessee, USA.; 5 Department of Medicine, Division of Allergy, Immunology, and Rheumatology, University of Rochester Medical Center, Rochester, New York, USA.; 6 South African Tuberculosis Vaccine Initiative, Institute of Infectious Disease and Molecular Medicine and Division of Immunology, Department of Pathology, University of Cape Town, Cape Town, South Africa.; 7 Biotechnology and Cell Signaling, CNRS UMR7242, Institut de Génétique et de Biologie Moléculaire et Cellulaire, CNRS UMR7104, INSERM U1258, Université de Strasbourg, Illkirch, France.; 8 Laboratory of Immunobiology and Genetics, Instituto Nacional de Enfermedades Respiratorias Ismael Cosio Villegas, Mexico City, Mexico.; 9 Tecnologico de Monterrey, Escuela de Medicina y Ciencias de la Salud, Mexico City, Mexico.; 10 Southwest National Primate Research Center, Texas Biomedical Research Institute, San Antonio, Texas, USA.

**Keywords:** Immunology, Infectious disease, Bacterial infections, Innate immunity, Tuberculosis

## Abstract

The ADP ribosyltransferases (PARPs 1–17) regulate diverse cellular processes, including DNA damage repair. PARPs are classified on the basis of their ability to catalyze poly-ADP-ribosylation (PARylation) or mono-ADP-ribosylation (MARylation). Although *PARP9* mRNA expression is significantly increased in progressive tuberculosis (TB) in humans, its participation in host immunity to TB is unknown. Here, we show that *PARP9* mRNA encoding the MARylating PARP9 enzyme was upregulated during TB in humans and mice and provide evidence of a critical modulatory role for *PARP9* in DNA damage, cyclic GMP–AMP synthase (cGAS) expression, and type I IFN production during TB. Thus, *Parp9*-deficient mice were susceptible to *Mycobacterium tuberculosis* infection and exhibited increased TB disease, cGAS and 2′3′-cyclic GMP-AMP (cGAMP) expression, and type I IFN production, along with upregulation of complement and coagulation pathways. Enhanced *M. tuberculosis* susceptibility is type I IFN dependent, as blockade of IFN α receptor (IFNAR) signaling reversed the enhanced susceptibility of *Parp9^–/–^* mice. Thus, in sharp contrast to PARP9 enhancement of type I IFN production in viral infections, this member of the MAR family plays a protective role by limiting type I IFN responses during TB.

## Graphical abstract

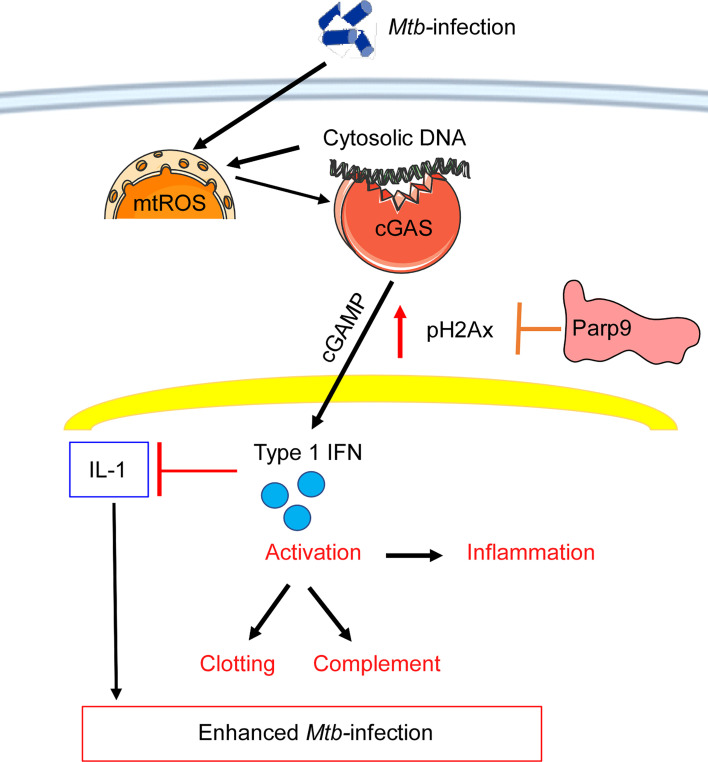

## Introduction

*Mycobacterium tuberculosis* infects approximately 25% of the world’s population and causes tuberculosis (TB), a leading infectious disease that results in approximately 1.3 million deaths annually. Although a majority of the infected individuals control *M. tuberculosis* infection (controllers, TB infection), approximately 10% of infected individuals develop active TB (progressors, active TB) (1). Importantly, there is a risk of disease reactivation in controllers and subsequent progression to TB disease, especially in immune-compromised and immune-suppressed individuals (2). *M. tuberculosis* is a pulmonary pathogen that establishes infection intracellularly within macrophages. However, the early macrophage-pathogen interactions within the lung remain poorly understood.

Our recently published work identified poly(ADP-ribose) polymerase 9 (*PARP9*) as a gene that is highly upregulated and acts as a correlate of risk for TB (3, 4). ADP-ribosylation is a form of posttranslational regulatory modification of proteins, nucleic acids, and metabolites that is catalyzed by ADP-ribosyltransferases (ARTs), which transfer ADP-ribose from NAD^+^ onto substrates. The 17 members of the mammalian ADP-ribosyl-transferases diphtheria toxin–like proteins (ARTDs; previously named PARP) family participate in various cellular processes such as DNA repair, genomic stability, and programmed cell death (5). The ARTD family of enzymes are classified according to their enzymatic activity as mono-ADP-ribosyltransferases (mono-ARTs) or poly-ADP-ribosyltransferases (poly-ARTs) (5). Although PARP9 was initially thought to be catalytically inactive, it appears to play a role in DNA damage repair and in driving immune responses including IFN-mediated antiviral defenses via an association with the E3 ligase DTX3L (6–9). In macrophages, PARP9 positively regulates proinflammatory cytokine production in response to IFN-γ stimulation by promoting phosphorylation of STAT1 (7).

Indeed, recent evidence indicates that PARP9 can act as a noncanonical RNA sensor that depends on the PI3K/AKT3 pathway and deltex 3/histone 2BJ to promote type I IFN production after viral infections with clinical implications (9, 10). Although the *PARP9* gene was found to be hypomethylated in patients with TB (11) and was identified as part of a 3-gene signature predicting progression to active TB in primates (12), the mechanistic and functional understanding of the role of PARP9 in the immunological processes occurring during active TB is unknown. Here, we show that PARP9 was upregulated in mouse and human TB. Using *M. tuberculosis* infection of *Parp9*-deficient mice, we demonstrate that, unlike a virus-induced effect, there was an important negative regulatory role for Parp9 in DNA damage, cyclic GMP–AMP synthase (cGAS) expression, and type I IFN production during TB. Of note, *Parp9*-deficient mice are susceptible to *M. tuberculosis* infection and exhibited increased TB pathology, elevated cGAS, 2′3′-cyclic GMP-AMP (cGAMP), and type I IFN expression, and upregulation of complement and coagulation pathways. Blockade of IFN α receptor (IFNAR) signaling reversed susceptibility in *Parp9^-/-^* mice, suggesting that the enhanced *M. tuberculosis* susceptibility was type I IFN dependent. Thus, although PARP9 mitigated the effects of TB infection, it did so by a mechanism opposite that of the type I IFN–promoting function of PARP9 in virus-infected cells.

## Results

### ARTDs are upregulated during M. tuberculosis infection across species.

**Figure 1:**
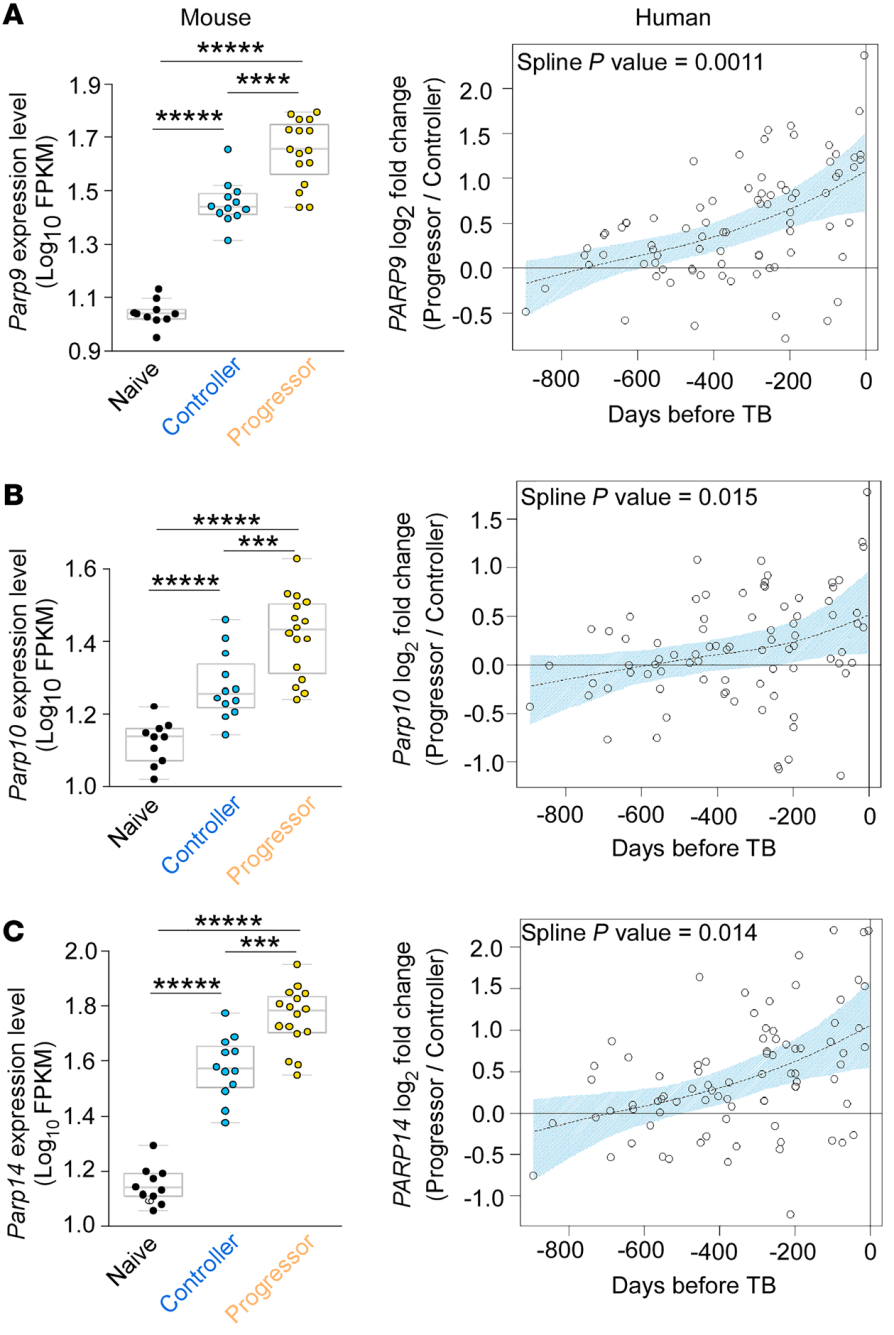
Transcriptional expression of MARylated PARP isoforms is upregulated in both human and mouse TB progressors. B6 or genetically diverse outbred (DO) mice were infected with *M. tuberculosis* HN878 (100 CFU) by the aerosol route. RNA isolated from lung homogenates of mice at 30 dpi was subjected to bulk RNA-Seq. (**A**–**C**) Ortholog gene expression of MARylated PARP isoforms across the ACS human blood transcriptomic profile from TB progressors and controllers, with the lung transcriptional profiles obtained from progressors and controller *M. tuberculosis*–infected DO mice (3). Human progressors, *n* = 46; human controllers, *n* = 107; mice, *n* = 39 (controller, *n* = 12; progressor, *n* = 16; naive, *n* = 10). All *P* values shown on the expression swarm plots represent FDR-corrected significance values for differential expression calculated by DESeq2. *****P < 10^–5^, ****P < 10^–4^, and ***P < 10^–3^. The human data used for comparisons were derived from Scriba et al. (4).

Our current understanding of the functional role of ADP-ribosylation and ARTDs in *M. tuberculosis* infection is limited. Thus, we compared the expression of ARTD family members in human blood transcriptomes from TB progressors and controllers (13) with the lung transcriptional profiles obtained from controllers and progressor *M. tuberculosis*–infected, genetically diverse outbred (DO) mice (3) (Figure 1, A–C, and Supplemental Figures 1–3; supplemental material available online with this article; https://doi.org/10.1172/JCI158630DS1). Of the *ARTD* members assessed, mRNA expression of the mono-ARTs *PARP9*, *PARP10*, and P*ARP14* was significantly upregulated in TB progressors in both humans and DO mice (controller vs. naive, progressor vs. naive, and progressor vs. controller) (Figure 1, A–C, and Supplemental Figure 1). In contrast, while other *ARTDs* showed similar expression levels, *TNKS1* (*PARP5*) and *PARP16* expression was significantly decreased in human TB progressors (Supplemental Figure 1). In *M. tuberculosis*–infected DO mice, mRNA levels for the poly-ADP-ribosylating (PARylating) *Parp* isoforms (*Parp1* and *Parp5*) were increased, whereas the expression of *Parp2* was decreased. Additionally, mouse MARylating *Parp* isoforms (*Parp3*, *Parp8*, and *Parp12)* were upregulated, while *Parp6* and *Parp16* isoforms were downregulated. Incidentally, whereas *PARP16* mRNA was consistently downregulated in both mice and human TB progressors, TB-associated changes in *PARP5* mRNA expression were not consistent in the mouse or human TB progressors. These results suggest that the cross-species *PARP* genes whose expression was induced to higher levels in both human and mouse TB progressors were MARylating *PARP9*, -*10*, and -*14* (Figure 1, A–C, and Supplemental Figure 1).

### PARP9 is expressed by macrophages within TB granulomas and required for M. tuberculosis control.

**Figure 2:**
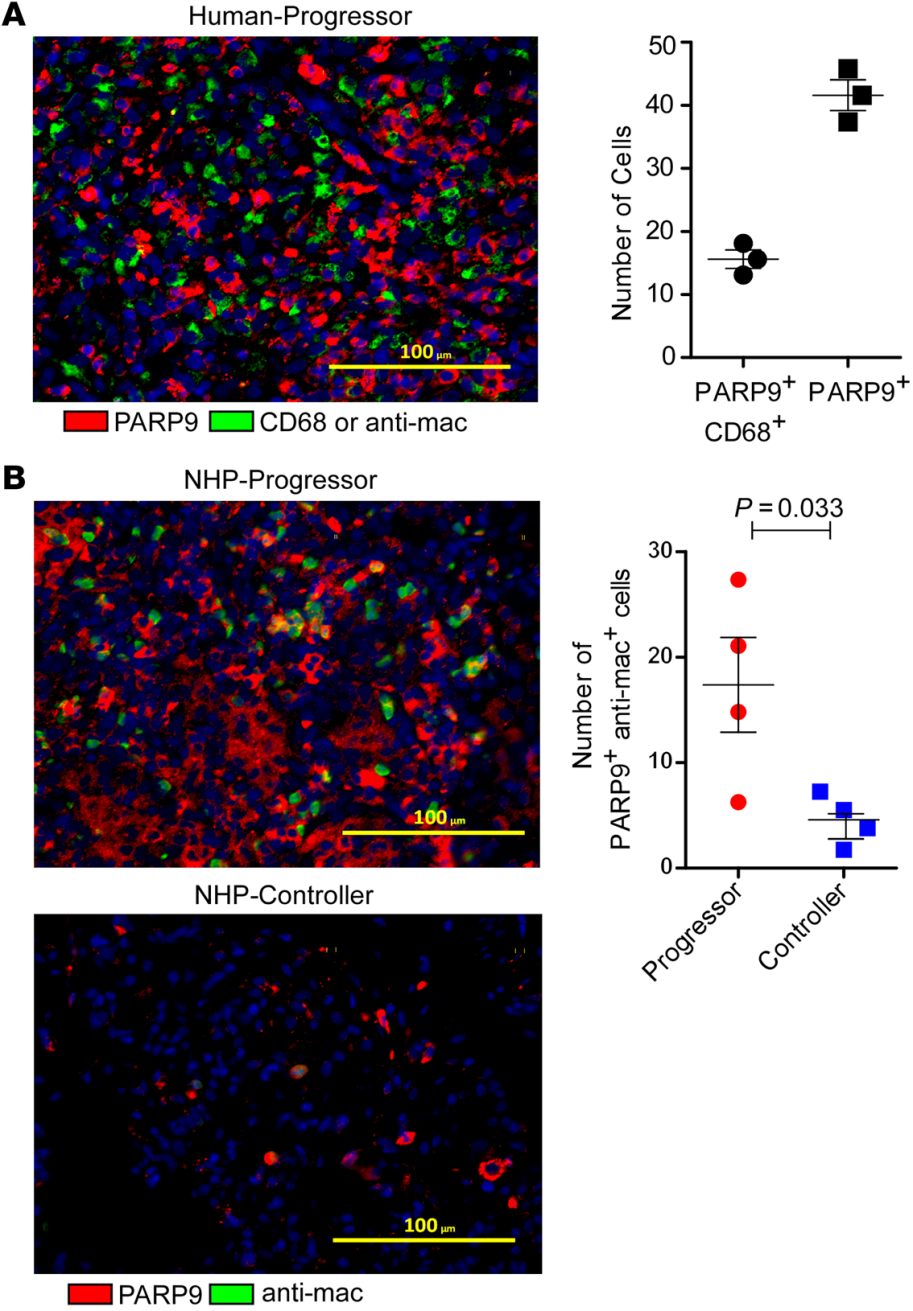
PARP9-expressing macrophages localize within TB granulomas. PARP9 protein expression in lung sections from (**A**) patients with active TB and (**B**) NHP progressors and controllers. Lung sections were stained for α-PARP9 antibody (red), human CD68^+^ macrophages (green), or NHP α-mac^+^ macrophages (green), and with DAPI (blue) to show nuclei. Representative photomicrographs of human (*n* = 3 granulomas) and NHP (*n* = 3 /group) lung tissues are shown. Scale bars: 100 μm. Original magnification, ×200. Data represent the mean ± SEM. *P* ≤ 0.05, by unpaired, 2-tailed Student’s *t* test.

As *PARP9* was induced in both mice and human TB progressors, we focused on the function of *PARP9* in TB. Lung macrophages are an essential source of PARP9 (14, 15). Consistent with this, CD68^+^ macrophages coexpressing PARP9 protein were detected in granulomas of *M. tuberculosis*–infected human and macaque progressors (Figure 2, A and B), but their numbers were significantly reduced within the lung granulomas of *M. tuberculosis*–infected macaque controllers (Figure 2B).

**Figure 3:**
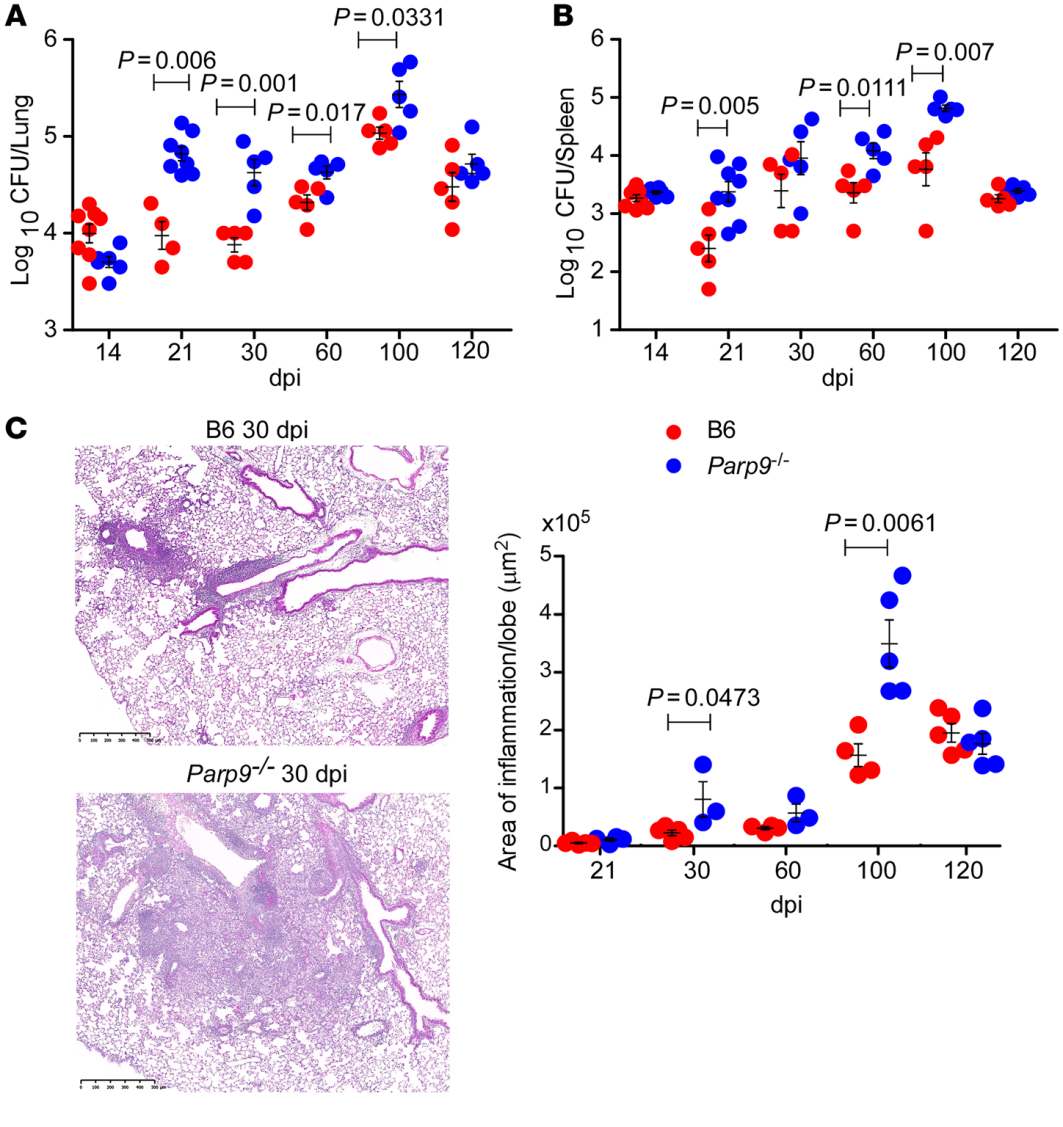
*Parp9^–/–^* mice exhibit increased early susceptibility to *M. tuberculosis* infection. B6 or *Parp9^–/–^* mice were infected with *M. tuberculosis* HN878 (100 CFU) by the aerosol route. Lungs and spleens were harvested on 14, 21, 30, 60, 100, and 120 dpi to assess the bacterial burden in (**A**) lungs and (**B**) spleen by plating. Lungs were harvested on 21, 30, 60, 100, and 120 after dpi and formalin fixed. (**C**) Representative images and the area of inflammation in each lobe by histological analysis. Scale bars: 500 μm. Original magnification, ×20. Data points represent the mean ± SEM of 1 of 2 individual experiments (*n* = 3–5, per time point per group). *P* *≤* 0.05, by unpaired, 2-tailed Student’s *t* test at different post-infection days.

To address the functional role of *Parp9* in *M. tuberculosis* infection, we next infected WT C57BL/6 (B6) and *Parp9*-deficient mice (*Parp9^–/–^*) with low doses of aerosolized *M. tuberculosis* HN878, a clinical *M. tuberculosis* strain. We found that *Parp9^–/–^* mice exhibited early increased *M. tuberculosis* CFU in the lungs when compared with B6 *M. tuberculosis*–infected lungs, and the increase in *M. tuberculosis* CFU was maintained until later time points (Figure 3, A and B). Bacterial dissemination is a critical indicator of disease progression. We detected increased *M. tuberculosis* CFU in the spleen at early (21 and 60 days post infection [dpi]) and late (100 dpi) time points in *Parp9^–/–^* mice. Coincident with increased lung *M. tuberculosis* CFU, *Parp9^–/–^* mice also exhibited enhanced inflammation with infection (Figure 3C). These results demonstrate that macrophages were a significant source of PARP9 inside TB granulomas and that PARP9 deficiency enhanced *M. tuberculosis* susceptibility and exacerbated lung inflammation.

### Parp9^–/–^ M. tuberculosis–infected mice exhibit increased myeloid cell accumulation and greater IFN responses.

**Figure 4:**
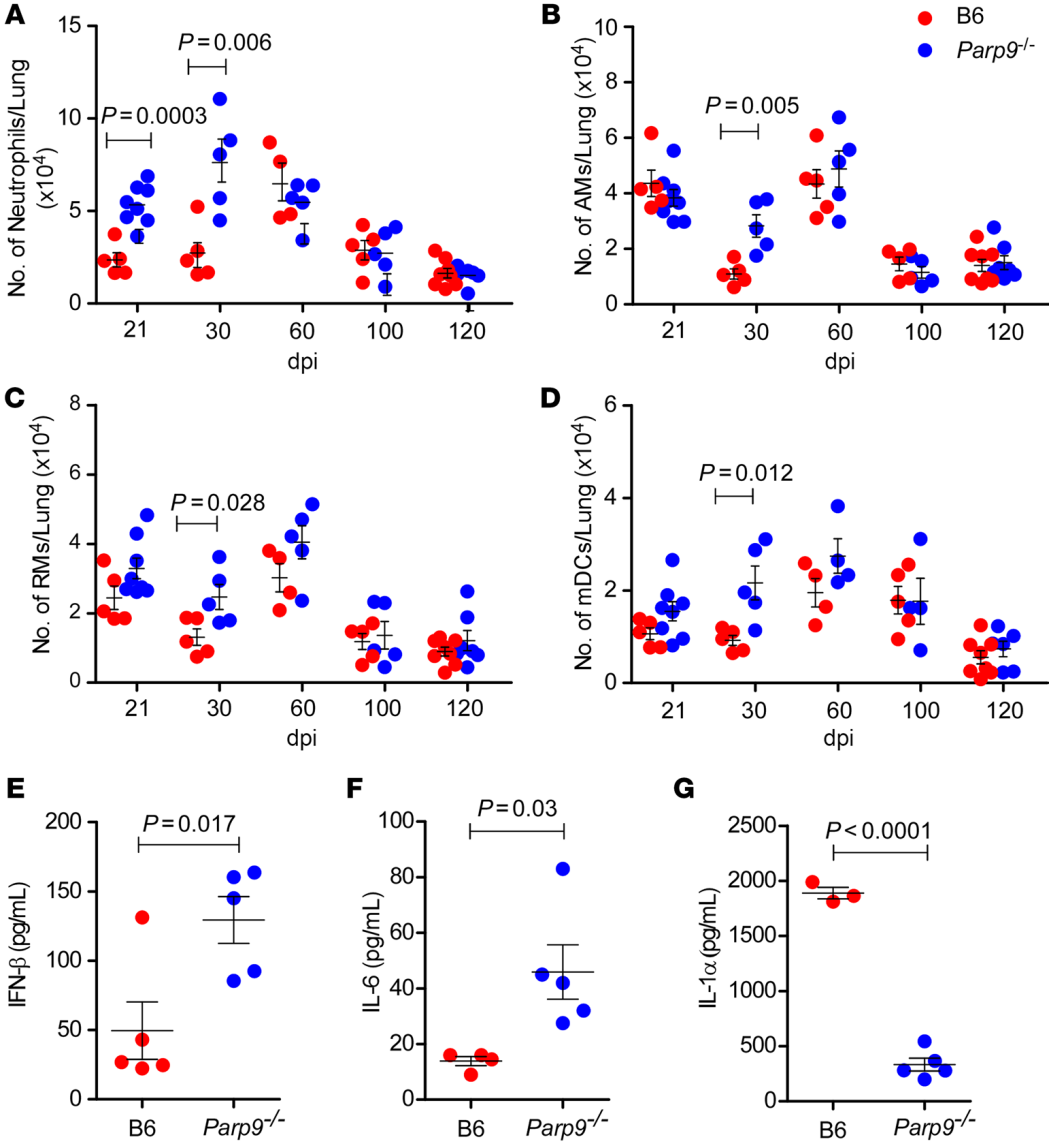
*Parp9* negatively regulates the accumulation of myeloid inflammatory cells and IFN responses during TB. B6 and *Parp9^–/–^* mice were infected with *M. tuberculosis* HN878 (100 CFU) by the aerosol route. (**A**) Neutrophils, (**B**) AMs, (**C**) RMs, and (**D**) mDCs were enumerated in the lungs of *M. tuberculosis*–infected mice by flow cytometry on 21, 30, 60, 100, and 120 dpi. (**E**–**G**) Cytokine production in lung homogenates from mice, collected at 30 dpi, was assessed by multiplex cytokine analysis. Data points represent the mean ± SEM of 1 of 2 individual experiments (*n* = 3–8 per time point per group). *P* ≤ 0.05, by unpaired, 2-tailed Student’s *t* test to determine significant differences between B6 and *Parp9^–/–^* mice.

PARP9 orchestrates type I IFN responses in viral infections (9, 10), but its functional role in IFN and myeloid responses during TB disease is unknown. Therefore, we assessed myeloid cell accumulation during early and chronic TB in B6 and *Parp9^–/–^ M. tuberculosis*–infected mice. We found that the increase in *M. tuberculosis* CFU in the lungs of *M. tuberculosis*–infected *Parp9^–/–^* mice coincided with an early and considerable accumulation of neutrophils, alveolar macrophages (AMs), recruited macrophages (RMs), and myeloid DCs (mDCs) (Figure 4, A–D). We assessed the cytokine responses in the lungs of *M. tuberculosis*–infected B6 and *Parp9^–/–^* mice during early disease, when the altered susceptibility was most pronounced. We observed a significant upregulation of IFN-β and IL-6 in lung homogenates from *M. tuberculosis*–infected *Parp9^–/–^* mice (Figure 4, E and F), contrasting with a reduction in the levels of the proinflammatory cytokine IL-1α (Figure 4G). Therefore, while studies have implicated PARP9 as a positive regulator of IFNs in viral infections (10, 16), our results suggest a protective function during TB for PARP9 mediated by limiting IFN production.

### Parp9 negatively regulates IFN-β expression in macrophages to limit M. tuberculosis susceptibility.

**Figure 5:**
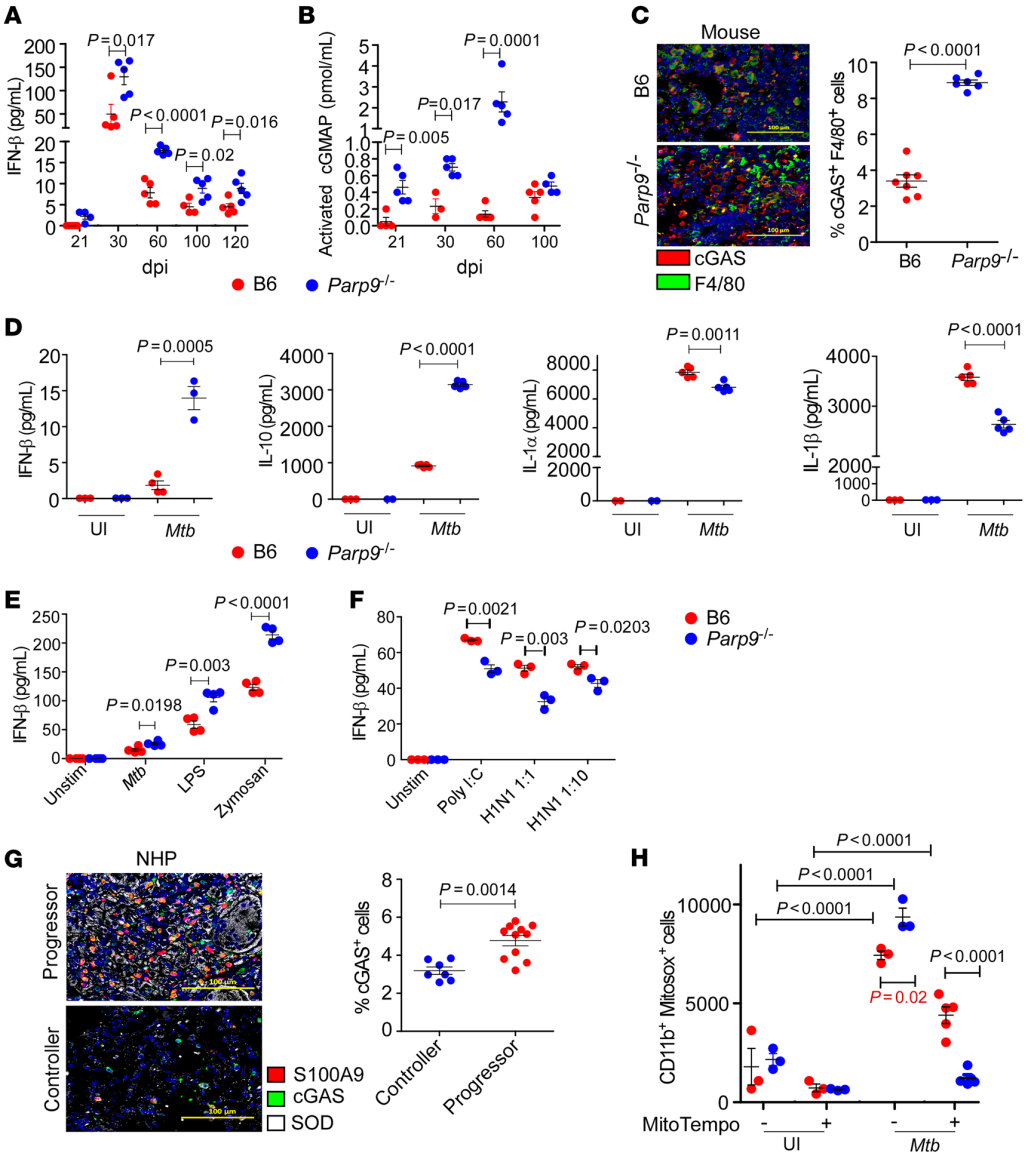
PARP9 regulates cGAS, mitochondrial oxidative stress, and type I IFN response. B6 and *Parp9^–/–^* mice were infected with *M. tuberculosis* HN878 (100 CFU) by the aerosol route. (**A**) IFN-β and (**B**) cGAMP were measured by ELISA in lung homogenates on 21, 30, 60, 100, and 120 dpi. (**C**) Representative immunofluorescence images and dot plot of cGAS protein expression (red) by F4/80^+^ macrophages (green) in lung sections from B6 and *Parp9^–/–^* mice. Scale bars: 100 μm. Original magnification, ×200. (**D**) IFN-β, IL-10, IL-1α, and IL-1β production was quantitated in supernatants from BMDM cultures after incubation with *M. tuberculosis* (*Mtb*) HN878 for 48 hours (MOI = 1). (**E** and **F**) IFN-β production in murine BMDMs cultured for 48 hours with *M. tuberculosis* HN878 (MOI = 1), the TLR agonists LPS or zymosan (25 μg/mL), and poly I:C or H1N1 (MOI = 1 or 10). (**G**) Representative images and dot plot of immunofluorescence detection of S100A9 (red) to show neutrophilic infiltration, cGAS (green), and SOD (white) in pulmonary granulomas from NHP TB controllers and progressors. Scale bars: 100 μm. Original magnification, ×200. (**H**) Mitochondrial oxidative stress in murine BMDM cultures after a 48-hour incubation with *M. tuberculosis* HN878 (MOI = 1). Data points represent the mean ± SEM of 1 of 2 individual experiments. *P* ≤ 0.05, by 2-tailed Student’s *t* test (**A**–**G**) and 1-way ANOVA with Tukey’s multiple-comparison test (**G**; Student’s *t* test for significance is denoted in red). *n* = 3–5 per group (**A**–**F** and **H**); *n* = 3 NHPs per group (**G**). UI, uninfected; Unstim, unstimulated.

Upon further analysis, we observed a significant increase in IFN-β and a corresponding decrease in IL-1β and IFN-γ (except at 60 dpi) protein levels in the lungs of *M. tuberculosis*–infected *Parp9^–/–^* mice across all time points tested (Figure 5A and Supplemental Figure 4, A and B). Therefore, we next sought to understand the underlying mechanism for enhanced IFN-β production in the absence of *Parp9* during *M. tuberculosis* infection. Upon recognizing pathogenic or self-DNA derived from the damaged cellular DNA, the cytosolic DNA sensor cGAS produces the second messenger cGAMP. It then activates stimulator of IFN genes (STING) signaling, which culminates in the production of type I IFNs and proinflammatory cytokines (17–19). Accordingly, we also observed a significant increase in 2′3′-cGAMP, a product synthesized by cGAS (Figure 5B), the expression of which was also increased in macrophages within granulomas of *M. tuberculosis*–infected *Parp9^–/–^* mice compared with B6 mouse lungs (Figure 5C). To further study the mechanisms by which Parp9 regulates cGAS and type I IFN responses, we then infected bone marrow–derived macrophages (BMDMs) from B6 and *Parp9^–/–^* mice with *M. tuberculosis* and measured cytokine responses. Consistent with the in vivo lung cytokine levels, we found that in vitro *M. tuberculosis*–infected *Parp9^–/–^* macrophages also produced significantly increased protein levels of IFN-β and IL-10 but produced substantially lower amounts of IL-1α and IL-1β proteins when compared with *M. tuberculosis*–infected B6 macrophages (Figure 5D). To further validate that *M. tuberculosis* HN878 and other TLR agonists induce similar responses, we compared IFN-β production in response to *M. tuberculosis* HN878 and known TLR2/-4 agonists such as LPS and zymosan. Our results showed that an IFN-β response was induced by *M. tuberculosis* HN878 and TLR agonists in B6 BMDMs but that the levels were higher in *Parp9^–/–^* BMDMs (Figure 5E). Consistent with published studies (10), treatment with the TLR3 agonist polyI:C and H1N1 virus resulted in a reduction in IFN-β levels in *Parp9^–/–^* BMDMs (Figure 5F), suggesting a differential role for Parp9 during *M. tuberculosis* infection.

Because type I IFN responses are associated with increased TB disease across species (3), we next assessed cGAS-expressing S100A9^+^ myeloid cells that also coexpressed superoxide dismutase (SOD) in the nonhuman primate (NHP) model of TB disease. SOD is an enzyme that acts as a scavenger of free radicals induced in response to *M. tuberculosis* infection (20). In the lungs of progressor macaques, we observed high levels of cGAS and SOD coexpression within S100A9^+^ myeloid cells, suggesting that activation of cGAS and SOD was associated with the induction of type I IFN responses. In contrast, very few cGAS^+^S100A9^+^ myeloid cells alone or coexpressing SOD were present in the lung granulomas of *M. tuberculosis*–infected controller macaques (Figure 5G). We assessed changes in the oxidative response of *Parp9^–/–^* mice infected with *M. tuberculosis* by evaluating mitochondrial oxidative stress and the ability of MitoTempo, a scavenger of mitochondrial oxidative stress, to reverse these effects. Our results showed enhanced mitochondrial oxidative stress in *Parp9^–/–^* BMDMs infected with *M. tuberculosis* HN878 compared with their B6 controls and demonstrated that incubation with MitoTempo reversed cellular mitochondrial oxidative stress (Figure 5H). These data together suggest that PARP9 negatively regulated the expression of cGAS, the production of cGAMP, and downstream IFN-β production in macrophages during *M. tuberculosis* infection.

We next examined lung transcriptional profiles in B6 and *Parp9^–/–^ M. tuberculosis*–infected mice and identified significantly differentially expressed genes between B6 and *Parp9^–/–^ M. tuberculosis*–infected lungs using DESeq2, version 1.4, with default settings (21) and a minimum *P* value significance threshold of 0.05 (after FDR correction for the number of tests) (22). Principal component analysis was also calculated using DESeq2 output with default settings, using the top 500 most variable genes. Our results show that transcriptional profiles of lung samples from uninfected B6 and *Parp9^–/–^* mice clustered together. However, the lung samples from *M. tuberculosis*–infected B6 and *Parp9^–/–^* mice did not cluster together despite clustering away from uninfected lungs (Supplemental Figure 5A). We found that 1,104 genes were exclusively upregulated in *Parp9^–/–^ M. tuberculosis*–infected lungs compared with B6 *M. tuberculosis*–infected lungs (Supplemental Figure 5B). Of note, these genes were represented by 188 pathways enriched in the lungs of *M. tuberculosis*–infected *Parp9^–/–^* mice compared with the lungs of *M. tuberculosis*–infected B6 mice. The top 20 pathways in the lungs of *M. tuberculosis*–infected *Parp9^–/–^* mice were associated with metabolism, fibrin clot formation, activation of the intrinsic pathway of fibrin clot formation, the complement cascade, and regulation of the complement pathway (Supplemental Figure 5C). Indeed, within the fibrin-associated coagulation pathways, we found increased expression of critical genes in this pathway, including coagulation factor II (*F2*) (prothrombin), which is proteolytically cleaved to form thrombin in the clotting process. Additional genes in the coagulation pathway included coagulation factor II (*F2*), VII (*F7*), VIII (*F8*), XI (*F9*), and XII (*F12*) (Supplemental Figure 5D), which encompass factors that function in both the intrinsic and the extrinsic pathways, namely tissue damage and blood trauma, respectively (23). Within the complement cascade, key induced genes included coagulation factor II (*F2*), complement 8 α (*C8a*), C8 β (*C8b*), and complement 8 γ (*C8g*), all of which were enhanced in *M. tuberculosis*–infected *Parp9^–/–^* mice (Supplemental Figure 6A). These genes function as the terminal component of the complement system and are part of both the complement membrane attack complex (MAC) and also crucial for MAC assembly (24). C-reactive protein (*Crp*), an acute-phase serum protein, binds to microbial polysaccharides or its ligands exposed on damaged cells, thereby activating the classical complement pathway and uptake by phagocytic cells (Supplemental Figure 6A). Finally, Complement receptor type 2 (*Cr2* or *Cd21*) (Supplemental Figure 6A) is part of the B cell receptor (*BCR*) coreceptor complex and activates the complement cascade to promote the differentiation of activated B cells into antibody-secreting plasma cells (25). Several members of the serine peptidase inhibitors (*serpine1C, -1A*, and -*1E* and *serpinC1*) were also elevated in *M. tuberculosis*–infected *Parp9^–/–^* mice (Supplemental Figure 6B). Upon recognizing *M. tuberculosis*, host serine proteases are activated, culminating in the assembly of complex, unstable proteases called C3/C5 convertases and activation of the complement pathway (26).

Thus, our results implicate *Parp9* as a negative regulator of type I IFN responses and, subsequently, of coagulation and complement pathways in *M. tuberculosis* infection. To validate this further, we constructed gene coexpression networks based on all RNA-Seq data analyzed in the human Adolescent Cohort Study (ACS) (13) and in the DO mouse immune correlates (3). Within each species data set, we calculated the Pearson correlation values between pairs of 2 genes and quantified the similarity of their expression (normalized gene expression data; log fragments per kilobase per million mapped reads [FPKM]) across all samples analyzed. Genes were included in the calculation only if we detected their expression in a minimum of 5 samples. In addition to *PARP9*, genes from pathways of interest were identified on the basis of the following Gene Ontology (GO) annotations (27) retrieved from Ensembl (28): GO: 0060337, type I IFN signaling pathway (18 mouse genes, 54 human genes); GO: 0007596, blood coagulation (74 mouse genes, 136 human genes); and GO: 0006958, complement activation, classical pathway (31 mouse genes, 24 human genes). The top genes coexpressed with PARP9 for the mouse and human data sets are shown in Tables 1 and 2, respectively. Indeed, in both the human and mouse analyses, the top coexpressed gene was deltex E3 ubiquitin ligase 3L (*DTX3L*), which binds to PARP9 and functions as an E3 ubiquitin ligase that selectively ubiquitinates histone H4 and protects cells against DNA-damaging agents (29). In viral infections, the interaction between PARP9 and DTXL3 appears to yield an antivirus effect by promoting the efficacy of type I IFN signaling (9, 10, 29). Other top genes coexpressed with *PARP9* include IFN regulatory factor 1 *(IRF1)*, which is involved in *STAT1* signaling. Basic leucine zipper ATF-like transcription factor 2 (*BATF2*) is known to associate with *IRF1* and *PARP14*, as well as with genes from the classic complement pathway including complement components *C1QA* and *C1QC* (Tables 1 and 2). Incidentally, in the human ACS progressor gene data set, 12 of the top 50 most highly *PARP9*-coexpressed genes were also related to type I IFN signaling and included *DTX3L*, IFN-induced protein with tetratricopeptide repeats 1, 2, 3, 5 (*IFIT*), *PARP14*, and 2′-5′-oligoadenylate synthetase 3 (*OAS3*).

We also analyzed the coexpression networks based on PARP9 and the pathways of interest in both mouse and humans (Supplemental Figure 7, A and B). In both networks, *PARP9* was coexpressed with *STAT1* and *SERPING1*. In mice, *Parp9* was connected to a network of coexpressed genes from all 3 pathways (Supplemental Figure 7A). In humans, it was connected to a network of coexpressed genes associated with type I IFN genes and *SERPING1* from the blood coagulation pathway (Supplemental Figure 7, B and C). These results suggest that *PARP9* expression in *M. tuberculosis*–infected mice and humans coincides with type I IFN, coagulation, and the complement cascade.

In addition, we examined the correlation between *PARP9* gene expression and relative protein abundance using a previously-published plasma protein microarray (30). This array consisted of 2,872 probes for 2,641 human proteins of interest for TB progression in the ACS cohort but omitted *PARP9* directly. Additionally, the RNA-Seq data from the ACS study described above and the protein array abundance values (30) were both available for 274 samples spanning both data sets. *PARP9* gene expression was correlated with protein abundance values using Pearson correlation values, as described above. The top proteins associated with *PARP9* gene expression from this analysis are included in Table 3.

We constructed a coexpression network on this protein array data set (Supplemental Figure 7C). In this network, *PARP9* still correlated strongly with STAT1 (the sixth-highest overall correlation value in Table 3). Although the gene coexpression network for the human genes showed the highest correlations between *PARP9* and genes from the type I IFN pathway, PARP*9* correlated most strongly with complement activation pathway genes in the protein array data.

### Exacerbated TB susceptibility in Parp9^–/–^ mice is mediated by type I IFN signaling.

**Figure 6:**
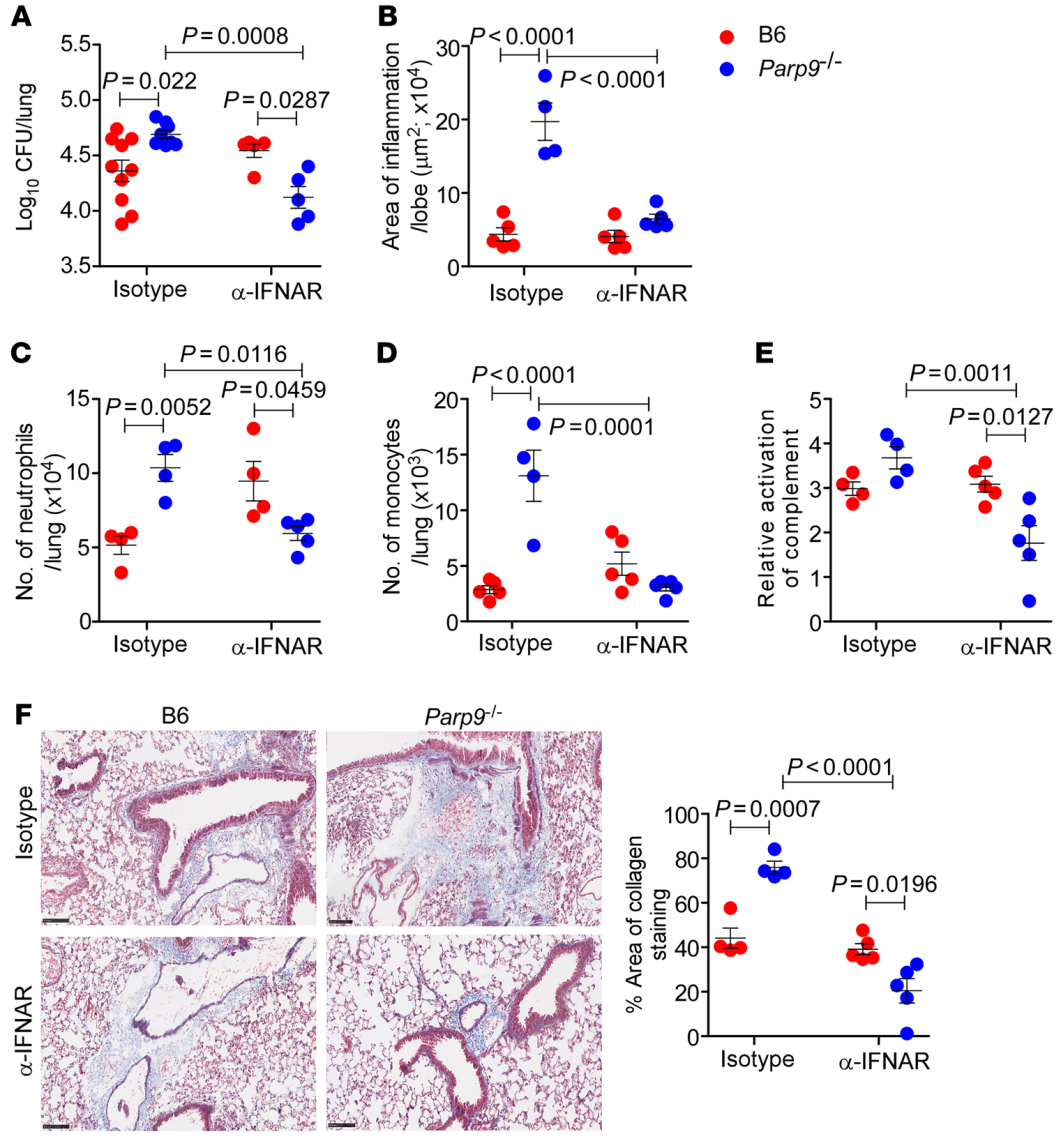
Enhanced TB susceptibility in *Parp9^–/–^* mice depends on type I IFN. B6 and *Parp9^–/–^* mice were infected with *M. tuberculosis* HN878 (100 CFU) by the aerosol route. Mouse α-IFNAR or mouse IgG1 isotype control antibody (500 μg/kg BW on days 7–9 and 250 μg/kg BW on alternate days), was administered i.p. in 300 μL PBS/mice until 30 dpi. Lungs were harvested on 30 dpi to assess the (**A**) bacterial burden and (**B**) area of inflammation. Lung single-cell suspensions were stained for FACS analysis to determine the number of (**C**) CD11b^+^ neutrophils and (**D**) monocytes. (**E**) Lung lysates were used to determine the relative activation of complement upon *M. tuberculosis* HN878 infection in B6 and *Parp9^–/–^* mice. (**F**) Representative photomicrographs of lung sections and corresponding dot plot. Lung sections were from *M. tuberculosis* HN878–infected B6 and *Parp9^–/–^* mice (*n* = 4–5 mice per group) treated with α-IFNAR or the isotype control and were stained by Carstair’s method for quantitation of collagen deposition and analyzed with ImageJ software (NIH). Scale bars: 100 μm. Original magnification, ×200. Data points represent the mean ± SEM, and analysis was done using 1-way ANOVA with Tukey’s multiple-comparison test (*n* = 4–10 mice per group). *P* ≤ 0.05 was considered significant.

**Figure 7:**
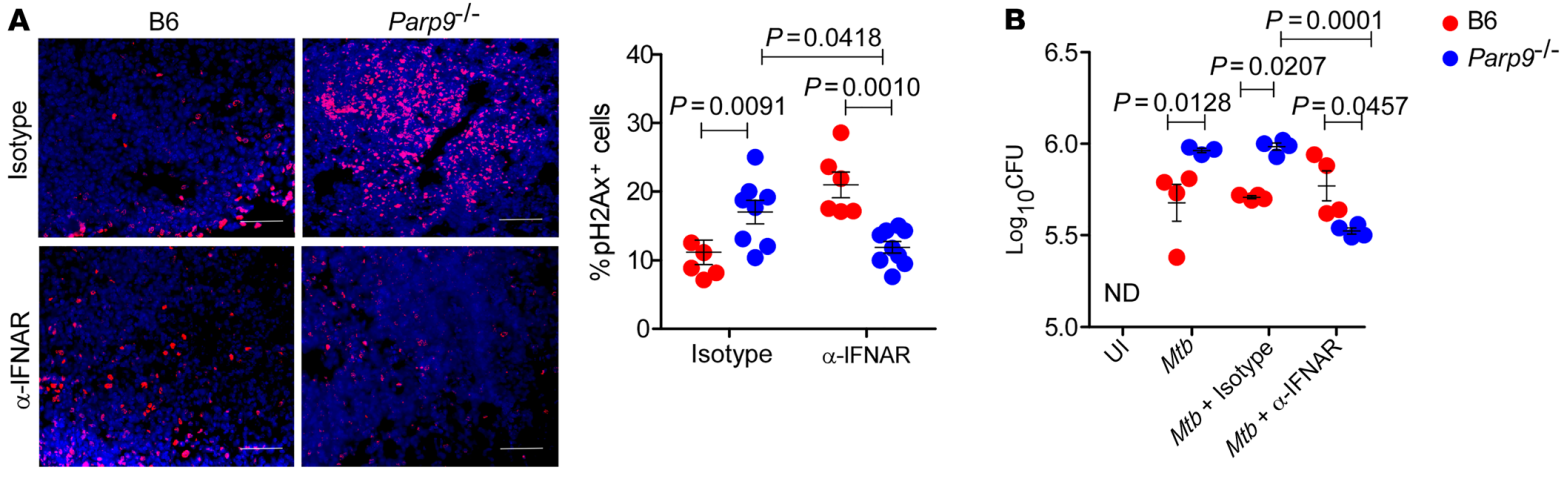
Enhanced DNA damage in *Parp9^–/–^* mice is type I IFN mediated. B6 and *Parp9^–/–^* mice were infected with *M. tuberculosis* HN878 (100 CFU) by the aerosol route. Mouse α-IFNAR or the mouse IgG1 isotype control antibody, at 500 μg/kg BW on days 7–9 and 250 μg/kg BW on alternate days, was administered i.p. in 300 μL PBS/mice until 30 dpi. (**A**) Representative images and corresponding dot plot of lung sections from *M. tuberculosis* HN878–infected B6 and *Parp9^–/–^* mice treated with α-IFNAR or an isotype control antibody. Lung sections were stained with α-pH2Ax antibody to assess DNA damage. The percentage of pH2Ax^+^ cells per high-powered field was quantitated manually. Scale bars: 100 μm. Original magnification, ×200. (**B**) Bacterial CFU in the in vitro–cultured *M. tuberculosis* HN878–infected BMDMs with or without α-IFNAR treatment. Data points represent the mean ± SEM, and analysis was done using 1-way ANOVA with Tukey’s multiple-comparison test (*n* = 3–5 mice per group). *P* ≤ 0.05 was considered significant.

We next tested the hypothesis that excess IFN-β expression mediates the increased TB susceptibility in *Parp9^–/–^ M. tuberculosis*–infected mice. Thus, we treated B6 and *Parp9^–/–^ M. tuberculosis*–infected mice with an IFNAR-blocking antibody (α-IFNAR) or a mouse IgG1 isotype control antibody (31). Treatment of *M. tuberculosis*–infected *Parp9^–/–^* mice with α-IFNAR decreased *M. tuberculosis* CFU, ameliorated lung inflammation, and impaired the accumulation of neutrophils and monocytes (Figure 6, A–D). Indeed, α-IFNAR treatment reversed the increased accumulation of complement factors (Figure 6E) and diminished collagen deposition in the lungs of *M. tuberculosis*–infected *Parp9^–/–^* mice (Figure 6F). As expected (31), treatment of B6 *M. tuberculosis*–infected mice with α-IFNAR did not affect *M. tuberculosis* control or any of the measured outcomes. These results provide experimental evidence that pathological hyper–type I IFN responses in the *Parp9^–/–^* mice enhanced *M. tuberculosis* susceptibility. To understand the mechanistic role of Parp9 in limiting type I IFN responses, we next studied whether the DNA damage response is differentially regulated in *Parp9^–/–^* mice and could contribute to cGAS/STING signaling and enhanced type I IFN production. The formation of double-stranded DNA breaks triggers the activation of many factors, including phosphorylation of the histone variant H2AX (32, 33). Therefore, we assessed the expression of pH2Ax in B6 and *Parp9^–/–^* mice with and without α-IFNAR treatment. *Parp9^–/–^* *M. tuberculosis*–infected mice exhibited increased pH2Ax expression within lung granulomas when compared with expression in B6 *M. tuberculosis*–infected lungs (Figure 7A). Furthermore, this increased expression of pH2Ax in *Parp9^–/–^* *M. tuberculosis*–infected lungs was type I IFN dependent, as the expression levels were reversed upon α-IFNAR blockade (Figure 7A). These finding translated in vitro to the reduction of *M. tuberculosis* bacterial CFU in BMDMs from *Parp9*^–/–^ mice that were treated with α-IFNAR (Figure 7B). Thus, we propose a model in which PARP9 induction during *M. tuberculosis* infection limits type I IFN, possibly via inhibition of a negative feedback loop, whereas during TB progression, PARP9 levels increase to modulate type I IFN signaling to limit *M. tuberculosis* susceptibility.

## Discussion

Mono- and poly-ARTs are important regulators of diverse cellular processes including DNA damage repair. Although PARP9 isoform expression is upregulated in human TB progressors (3, 13), its functional role in TB immunity is unknown. Here, we report an increase in *PARP9* mRNA expression during murine and human TB. We uncovered an unexpected negative regulatory role for *Parp9* in cGAS and type I IFN signaling during TB in *Parp9^–/–^* mice. In a surprising paradox, *Parp9^–/–^* mice were susceptible to *M. tuberculosis* infection and exhibited exacerbated TB disease severity, which coincided with increased DNA damage, increased expression of cGAMP, cGAS, and type I IFN–regulated genes, as well as upregulation of complement and coagulation pathways. The increased *M. tuberculosis* susceptibility in *Parp9^–/–^* mice was type I IFN dependent, as blockade of IFNAR signaling reversed susceptibility to TB. Therefore, our studies describe what we believe to be a novel type I IFN counterregulatory role for PARP9 that is opposite the known role of PARP9 in promoting type I IFNs and antiviral functions (9, 10). Together, these results provide evidence of a dual role for PARP9 in regulating the type I IFN pathway and thereby influencing TB susceptibility, thus calling for caution when considering ARTDs such as PARP9 as new therapeutic targets to improve host TB immunity.

A transcriptional type I/II IFN–responsive signature is present in patients with TB (13), the lungs of TB progressors, TB animal models (3), and in the blood of susceptible *M. tuberculosis*–infected mice (34). Indeed, deficiency of type I IFN signaling reverses exacerbated disease susceptibility in inbred mice susceptible to TB (34). Type I IFNs exert multiple regulatory effects, including induction of the antiinflammatory cytokine IL-10 and limitation of IL-1 and IL-12 in *M. tuberculosis–*infected macrophages (35–37). In addition, type I IFNs mediate exacerbated neutrophil extracellular trap (NET) formation and myeloid cell accumulation, while promoting mycobacterial growth (34, 38). Thus, our results showing increased type I IFN responses, increased myeloid cell accumulation, decreased IL-1β production, and increased *M. tuberculosis* burden as well as TB disease in *Parp9^–/–^* mice are consistent with the idea that PARP9 provides a protective role by limiting type I IFNs.

The list of newly identified IFN induction signaling pathways recently expanded with tripartite motif–containing (Trim) 30α (Trim30α) (37). Trim30α augments the type I IFN response, however, overexpression of Trim30α promotes the degradation of STING (37). TRIM30α is thus a crucial negative feedback regulator of the type I IFN response (37). Significant in this context is that next only to *Dtxl3*, *Trim30a* is the gene with the highest coexpression with *Parp9* across the mouse RNA-Seq samples analyzed. Therefore, it is likely that, concurrent to cGAS and mitochondrial oxidative stress–driven induction of type I IFN, *Parp9* also plays a role in *Trim30a*-mediated control of type I IFN, thus establishing a role for *Parp9* in modulation of type I IFN production.

*M. tuberculosis* infection has been shown to induce considerable DNA damage in host cells (39). PARP9 was initially thought to lack the intrinsic capacity to catalyze ADP-ribosylation. However, later evidence indicated that a heterodimer of PARP9 with the protein DTX3L displays MARylating activity (9). MARylation of a protein by PARP9 leads to ubiquitination by DTX3L (9). The PARP9/DTX3L heterodimer also ubiquitinates histone H4 and protects cells against DNA-damaging agents (9). Our results show that DTX3L was the most highly coexpressed gene with PARP9 in human and mouse TB progressors. Thus, it is likely that PARP9-DTX3L interactions play a functional role in protecting host cells against DNA damage and limiting type I IFNs in TB, possibly via a feedback loop involving the induction of *IRF2*, *IRF9*, and *TRIM30a*. Our notion is supported by our finding that the indicator of DNA damage, pH2Ax expression, was upregulated in *Parp9^–/–^* mice but was significantly reduced upon blocking type I IFN production. Thus, we propose that in the absence of PARP9 in *M. tuberculosis*–infected mice, the compromised DNA repair promoted oxidative stress, driving cGAS-mediated type I IFN induction, which was unregulated. In progressors (humans and macaques) as well as DO mice, increased expression of PARP9 could occur to regulate early increases in *M. tuberculosis* infection–induced oxidative stress and type I IFN. However, enhanced PARP9 expression occurred concurrently with increases in PARP14 as well as other PARylating enzymes. DNA processing and repair factors, if uncontrolled, can themselves generate DNA damage and the resultant increases in oxidative stress, cGAS expression, and type I IFN responses (40, 41). Therefore, it is likely that the PARP9-induced regulatory effect on type I IFN production could be thwarted by the responses occurring due to other enhanced PARP isoforms. However, on the basis of the data from *Parp9^–/–^* mice with enhanced IFN responses, we propose a model in which *PARP9* (and likely *DTXL3*) expression in TB progressors is protective and that increased expression is a reflection of its function in counterregulating and limiting excessive type I IFN production. In other words, in humans and NHPs, *M. tuberculosis* infection drives PARP expression as a protective mechanism, but uncontrolled PARP activity actually contributes to disease progression.

Recently, several studies have provided evidence for the role of type I IFNs in activation of the complement cascade and proposed the potential for targeting and selectively inhibiting the complement system as a therapeutic option for bacterial, viral, parasitic, and other inflammatory diseases (42–45). Activation of complement pathways results in the formation of bioactive molecules, mainly C3a and C5a, which act as potent chemoattractants and contribute to the enhanced migration and recruitment of inflammatory cells, the activation of phagocytic cells, and the release of proinflammatory agents and free radicals (46). Thus, type I IFN–driven activation of complement may function as a link to the inflammatory changes observed in *Parp9^–/–^* mice. Indeed, TB progressors have enhanced complement activation (4), supporting the possibility that PARP9-regulated type I IFN production influences TB pathogenesis in humans and NHPs. However, the intricate signaling mechanisms and agents mediating these responses deserve further detailed analysis and create an attractive area of research, especially if this axis is an amenable target to improve TB therapeutics.

PARP9 in viral infections have been associated with promoting type I IFN–mediated antiviral immunity (7, 9, 10) However, a key finding in our in vivo and in vitro studies using a highly relevant bacterial pathogen such as *M. tuberculosis* showed a contrasting role for PARP9 in *M. tuberculosis* host immunity. The differential molecular mechanisms that viral and bacterial determinants utilize to induce or suppress the host’s immune machinery could explain these contrasting pathogen-specific effects. For instance, it is known that the Parp9-Dtx3L complex can directly catalyze MARylation of viral proteins, leading to their degradation (9). The current lack of knowledge about the molecular identity of bacterial cell wall components that are targets of the PARP9 complex limits our understanding of why PARP9 has an opposite role in viral and bacterial infections.

Our findings therefore establish a role for *PARP9* that is contrary to its role in viral infections. Therefore, while *PARP9* is undoubtedly a biomarker for TB disease progression, its role as a target for host-directed therapeutics is still unclear. Several FDA-approved PARP inhibitors for patients with cancer show promising modulatory features in vitro and in vivo. However, in light of our current findings, caution is advocated when choosing these agents as possible therapeutics for TB, given their potential role in feedback loop mechanisms that might have far-reaching implications for the host’s physiological state during TB disease. Nevertheless, understanding the functional mechanisms of PARP family host-directed therapeutic biomarkers such as PARP9 is crucial for distinguishing between those that might prove beneficial and those that might be harmful.

Our studies indicate that PARP9 is an early regulator of *M. tuberculosis* infection–induced pathogenesis and a critical negative regulator of cGAS-type I IFN responses. Indeed, identification of a negative regulatory role of PARP9 in type I IFN induction sheds further light on the IFN regulatory network and represents an attractive target for improving therapeutic approaches in TB.

## Methods

### Mice

Generation of *Parp9^–/–^* mice (on a C57BL/6 background) was done as previously described (47). C57BL/6 (B6) mice were purchased from The Jackson Laboratory. All mice were bred in the animal facility at Washington University School of Medicine. Mice were age and sex matched and used between the ages of 6 and 8 weeks.

### M. tuberculosis infection

#### M.

*tuberculosis* HN878 was cultured in Proskauer Beck medium containing 0.05% Tween-80 to reach mid-log phase and frozen in 1 mL aliquots at –80^o^C until use. Mice were aerosolized with approximately 100 CFU of the *M. tuberculosis* HN878, using a Glas-Col airborne infection system (48). The right lung lobes were harvested and homogenized at 14, 21, 30, 60, 100, and 120 dpi. Ten-fold serial dilutions of homogenates were plated on Petri dishes containing 7H11 agar solid medium (BD Biosciences) and CFU counted after 2–3 weeks.

### H1N1 infection

Influenza A/PR/8/34 (influenza H1N1) stock (2.6 × 10^7^ PFU/mL), propagated in chicken eggs as previously described (49), was a gift from Radha Gopal (Department of Pediatrics, UPMC Children’s Hospital of Pittsburgh, Pittsburgh, Pennsylvania, USA). For in vitro infections, H1N1 was used at MOIs of 1:1 and 1:10.

### Flow cytometric staining of lung single-cell suspensions

B6 and *Parp9^–/–^* mice were euthanized with CO_2_, and the left lower lobe was isolated and perfused with heparin in saline. Single-cell suspensions of lung cells from untreated or *M. tuberculosis*–infected mice were isolated as previously described (48). Briefly, lungs were minced and incubated in collagenase/DNAse for 30 minutes at 37°C. Lung tissue was then filtered through a 70 μm nylon screen to obtain a single-cell suspension. Erythrocyte-free cell suspensions were further washed and resuspended in complete DMEM (high-glucose/10% FBS/ 1% penicillin-streptomycin) for flow cytometric staining. The following fluorochrome-conjugated antibodies were used for cell-surface staining: CD11b-APC (clone: M1/70, Tonbo Biosciences); CD11c-PeCy7 (clone: HL3, BD Biosciences); and GR-1 PE (clone: RB6-8C5, Tonbo Biosciences). Samples were acquired on a 4-laser BD Fortessa Flow Cytometer, and the analysis was performed using FlowJo (Treestar) software. Alveolar macrophages (CD11c^+^CD11b^–^), neutrophils (CD11b^+^CD11c^–^Gr-1^hi^), monocytes (CD11b^+^CD11c^–^Gr-1^med^), and recruited macrophages (CD11b^+^CD11c^–^Gr-1^lo^) were defined as previously reported by our group (50).

### Histological and immunohistochemical analysis

The left upper lobe was collected for determination of inflammation by histological analysis. The lobes were infused with 10% neutral buffered formalin and embedded in paraffin. Lung sections (5 μm thick) were stained with H&E or Carstair (51, 52) to visualize fibrillary collagen. Images were captured using the automated Nanozoomer digital whole-slide imaging system (Hamamatsu Photonics). Regions of inflammatory cell infiltration were delineated using NDP view2 software (Hamamatsu Photonics), and the percentage of inflammation was calculated in relation to the total lung area of each section. All scoring was conducted in a blinded manner, using 3–5 mice per group. Formalin-fixed, paraffin-embedded lung sections were subjected to immunohistochemical analysis using the following antibodies: CD68 (mouse α-CD68, clone PG-M1, Genetex, catalog GTX73723, RRID: AB_375099); PARP9 (rabbit α-PARP9, Thermo Fisher Scientific, catalog PA5-48254, RRID:AB_2633712); mouse α–human macrophages (α–human macrophages/monocytes/granulocytes, Bio-Rad, catalog MCA874G, RRID:AB_321963); cGAS (rabbit α-C6orf150 [cGAS], Thermo Fisher Scientific, catalog PA5-76367, RRID: AB_2720094); S100A9: (goat α–mouse S100A9, R&D Systems, catalog AF2065, RRID: AB_2184263); SOD (mouse α-SOD, clone 2A1, Abcam, catalog ab16956, RRID: AB_302569); mouse F4/80 (rat α–mouse F4/80, clone Cl:A3-1, Bio-Rad, catalog MCA-497R, RRID: AB_323279); and polyclonal rabbit α–histone H2A.X (Genetex, catalog GTX108272, RRID: AB_1950472).

### In vitro M. tuberculosis infection of BMDMs

Bone marrow cells (1 × 10^7^), flushed off from the femur and tibia of C57BL/6 and *Parp9^–/–^* mice, were plated in 10 mL complete DMEM (cDMEM) supplemented with 20 ng/mL recombinant mouse granulocyte-macrophage colony-stimulating factor (rmGM-CSF) (catalog 315-03, Peprotech). Cells were then cultured at 37°C in 5% CO_2_. cDMEM (10 mL) containing 20 ng/mL rmGM-CSF was added to the cultures, and adherent cells were collected on day 7 as BMDMs (53). BMDMs were infected with either *M. tuberculosis* HN878 or H1N1 at an MOI of 1:1 or 1:10. A TLR agonist was used to stimulate in vitro BMDM cultures included LPS (MilliporeSigma, 25 μg/mL), zymosan, or poly I:C (InvivoGen, 25 μg/mL). In some cases, macrophages were treated with α-IFNAR (clone MAR1–5A3, Bio X Cell) or an isotype control (MOPC-21, Bio X Cell) at 25 μg/mL, on day –1 and 3 dpi. Infected macrophages were washed rigorously with sterile PBS to remove nonphagocytosed *M. tuberculosis*, and then lysed with 0.05% sterile SDS for 5 minutes, followed by plating in serial dilutions on 7H11 agar plates to estimate intracellular CFU.

### Detection of protein and intermediates of the type I IFN pathway

#### Cytokine quantification.

Cytokines were measured in total lung homogenates and cell culture supernatants with Milliplex Multiplex Assays (MilliporeSigma) or the human/mouse IFN-β Immunoassay kit (R&D Systems).

#### Detection of cGAMP.

Modulation of cGAS activity in total lung cell homogenates was performed using the 2′3′-cGAMP ELISA kit (Cayman Chemical) according to the manufacturer’s instructions.

#### Detection of complement activation.

Activation of the classic complement pathway was detected by assessing the terminal C5b-9 complex in lung homogenates using a mouse classical complement pathway assay ELISA kit (Hycult Biotech)

### Detection of mitochondrial oxidative stress

BMDMs (4 × 10^5^ cells/well), after 6 days of differentiation, were plated in 24-well plates with cDMEM without antibiotics and incubated with *M. tuberculosis* HN878, LPS (from *Escherichia coli* O55:B5, catalog L2880, MilliporeSigma), or zymosan (catalog L4250, MilliporeSigma) at 25 μg/mL and incubated at 37°C, 5% CO_2_ for 48 hours. The media were removed, and macrophages were lifted off the culture plates using gentle pipetting after a 5-minute incubation at 4°C in ice-cold PBS without Ca^+^ and 2 mM EDTA. Macrophages were centrifuged in 96-well, U-bottomed plates at 300*g* for 6 minutes at 4°C before staining with the surface marker CD11b. Cells were then incubated with 2.5 mM MitoSOX Red (MSR) (Invitrogen, Thermo Fisher Scientific) in staining media for 10 minutes at 37°C, 5% CO_2_, followed by LIVE/DEAD Aqua staining for 10 minutes at 4°C. Macrophages were washed and subsequently fixed with 1% formalin in flow staining medium, and data were acquired on a BDX20 (BD Biosciences). Live nondebris singlets were analyzed for fluorescence in the MSR channel to quantify mROS.

### RNA-Seq and processing

Human data for the blood RNA signature were retrieved from a previous study (13). The significance values for association with progressors or controllers were aligned to mouse genes using these human gene matches to the mouse genome as detailed previously (3). Read counts, relative gene expression levels, gene annotations, and differential expression data for the mouse genes and their corresponding macaque genes and the alignment to the human data set were as explained earlier (3). Mouse lung tissues (B6/*Parp9^-/-^)* were homogenized and snap-frozen in RLT buffer, and DNase-treated total RNA was extracted using the QIAGEN RNeasy Mini kit (QIAGEN) (3). Library preparation was performed with 500 ng to 1 μg total RNA. rRNA was removed by an RNase-H method using RiboErase kits (Kapa Biosystems). mRNA was then fragmented in reverse transcriptase buffer and heated to 94°C for 8 minutes. mRNA was reverse-transcribed to yield cDNA using SuperScript III RT enzyme (Life Technologies, Thermo Fisher Scientific, according to the manufacturer’s instructions) and random hexamers. A second strand reaction was performed to generate ds-cDNA. cDNA was blunt-ended, had an A base added to the 3′ ends, and then had Illumina sequencing adapters ligated to the ends. Ligated fragments were then amplified for 12–15 cycles using primers incorporating unique dual index tags. Fragments were sequenced on an Illumina NovaSeq 6000 using paired-end reads extending 150 bases. On average, 65 million reads per sample were sequenced.

After adapter trimming using Trimmomatic, version 0.39 (54), sequenced RNA-Seq reads were aligned to the *Mus musculus* genome (GRCm39, Ensembl release 103) (55) using the STAR aligner, version 2.7.5b (56) (2-pass mode, basic). All raw RNA-Seq fastq files were uploaded to the NCBI’s Sequence Read Archive (SRA) (57) (https://www.ncbi.nlm.nih.gov/sra; BioProject accession no. PRJNA753056). Complete sample metadata and accession information are provided in Supplemental Table 1 (BioProject accession no. PRJNA753056). Read fragments (read pairs or single reads) were quantified per gene per sample using feature Counts, version 1.5.1 (58).

Significantly differentially expressed genes among naive, controller, and progressor sample sets were identified using DESeq2, version 1.4.5 (21), with default settings and a minimum *P* value significance threshold of 0.05 (after FDR correction for the number of tests) (22). Principal component analysis was also done using DESeq2 output (default settings, using the top 500 most variable genes). Fragments per kilobase of gene length per million reads mapped (FPKM) normalization was performed using DESeq2-normalized read counts. Read counts, relative gene expression levels, gene annotations, and differential expression data (4 comparisons) for every mouse gene are provided in Supplemental Table 2 (BioProject accession no. PRJNA753056). Pathway enrichment analysis among differentially expressed gene sets of interest was performed for Reactome pathways (59) on the WebGestalt web server (60) (*P* ≤ 0.05 after FDR correction; minimum of 3 genes per term), using a background of all protein-coding genes. Coexpression networks based on *PARP9* and the pathways of interest were built using Cytoscape (version 3.8.2, “Edge-weight spring-embedded layout” algorithm, plus a manual adjustment for visibility) (61), connecting genes with a minimum Pearson coexpression value of 0.85, corresponding to 2.5 and 2.9 SDs above the mean coexpression values for mice and humans.

In addition, the correlation was examined between *PARP9* gene expression and relative protein abundance, using a previously-published plasma protein microarray (30). A coexpression network was also constructed based on this protein array data set. Because of the additional noise and variability inherent to protein arrays, a minimum Pearson correlation of 0.6 was applied to visualize a similar number of connections to the gene expression network. Additionally, given the variability between RNA-Seq and protein arrays, a minimum coexpression value of 0.3 was applied between the *PARP9* gene expression values and the protein abundance values.

### Statistics

For all representative experiments, data were reproduced at least once. Differences between the means of 2 groups were analyzed using a Student’s *t* test, and for multiple-group comparisons, 1-way ANOVA with Tukey’s post test was applied. Statistical analysis was performed using GraphPad Prism 8 (GraphPad Software). Grubb’s outlier analysis was used to exclude any data points. A *P* value of 0.05 or less was considered significant. Data in the figures are presented as the mean ± SEM. For RNA-Seq analysis of human and mouse samples, significantly differentially expressed genes among naive, controller, and progressor sample sets were identified using DESeq2, version 1.4.5, with default settings (21) and a minimum *P* value significance threshold of 0.05 (after FDR correction for the number of tests) (22). Pearson’s coexpression method was used to identify the genes and proteins with the highest coexpression with PARP9. The coexpression network was built using Cytoscape software.

### Study approval

All experiments using animals were conducted in accordance with IACUC guidelines of Washington University in St. Louis and were approved under protocol 20160129. Protocols involving the use NHPs were approved by the IACUC of the Tulane National Primate Research Center. The human samples were obtained with informed consent from the Tuberculosis Outpatient Clinic at the National Institute of Respiratory Diseases (INER) in Mexico City, before α–*M. tuberculosis* treatment, and the protocol for their use was approved by the INER IRB. No compensation was provided to patients. All experiments were performed in accordance with the protocols.

## Author contributions

SAK conceptualized the study. ST, MA, BAR, SHC, JRM, SKM, VS, AG, and MB performed mouse experiments and analysis. MSK, BAR, MM, and TJS were responsible for analysis of human samples. ST, MA, and SAK wrote the original draft of the manuscript. ST, MA, BR, MB, SHC, JRM, SKM, VS, MM, DK, JZ, TJS, and SAK reviewed and edited the manuscript. MM, DK, TJS, and SAK acquired funding. SAK supervised the study.

## Supplementary Material

Supplemental data

## Figures and Tables

**Table 3:**
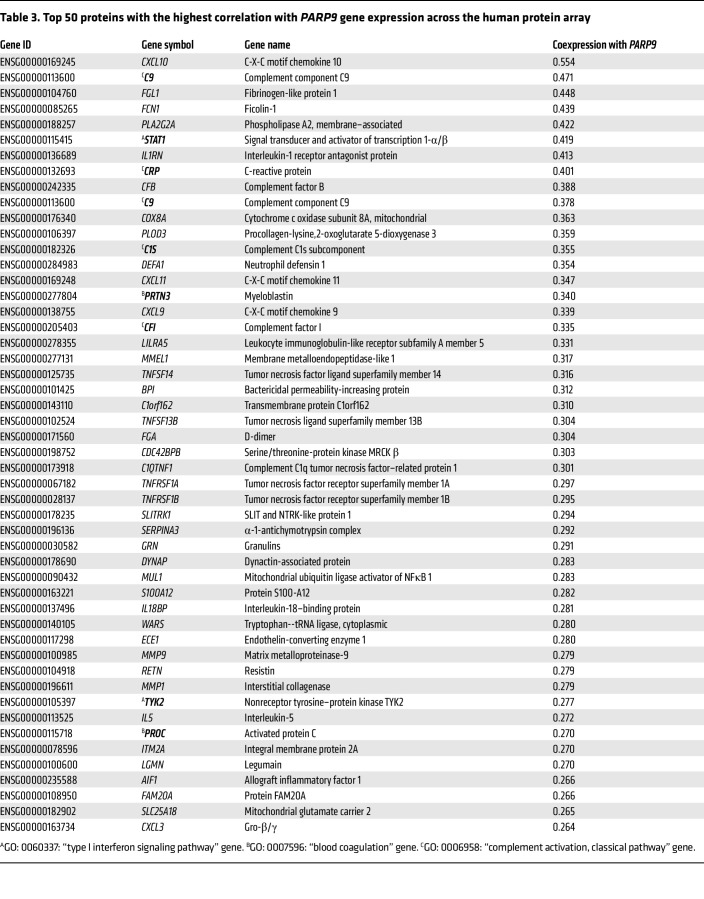
Top 50 proteins with the highest correlation with *PARP9* gene expression across the human protein array

**Table 2:**
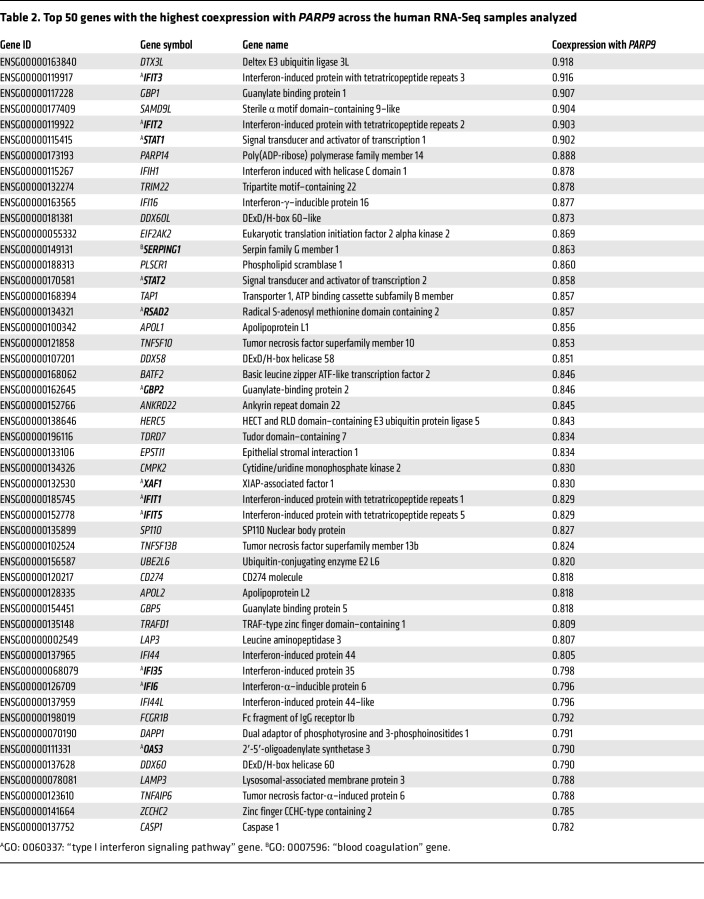
Top 50 genes with the highest coexpression with *PARP9* across the human RNA-Seq samples analyzed

**Table 1:**
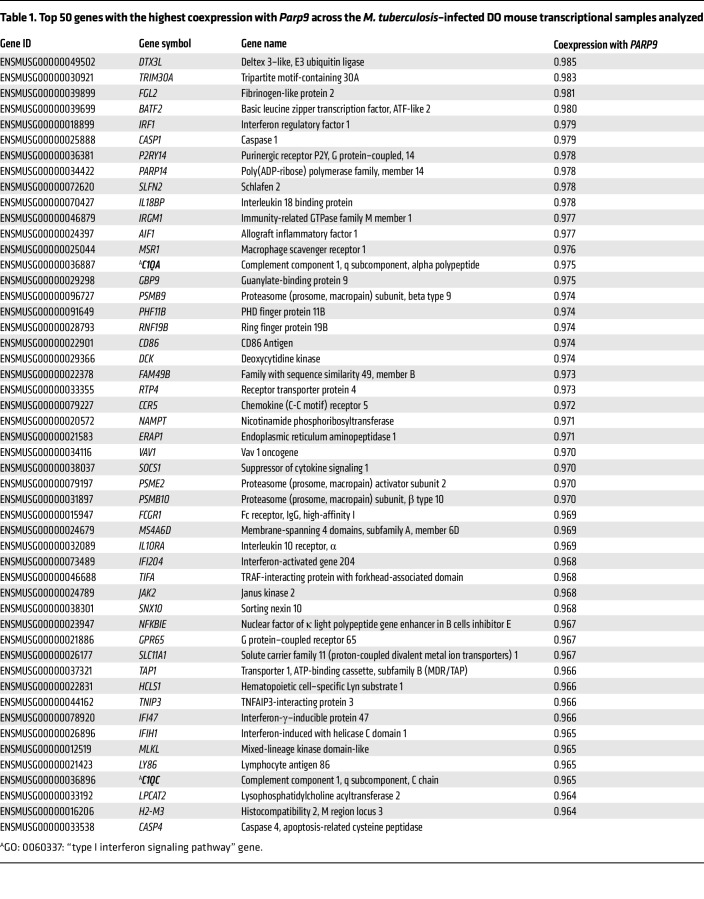
Top 50 genes with the highest coexpression with *Parp9* across the *M. tuberculosis*–infected DO mouse transcriptional samples analyzed

## Version 1 05/18/2023

In-Press Preview

## Version 2 06/15/2023

Electronic publication

## References

[1] Koegelenberg CFN (2021). Tuberculosis: the past, the present and the future. Respiration.

[2] Sandgren A (2016). Identifying components for programmatic latent tuberculosis infection control in the European Union. Euro Surveill.

[3] Ahmed M (2020). Immune correlates of tuberculosis disease and risk translate across species. Sci Transl Med.

[4] Scriba TJ (2017). Sequential inflammatory processes define human progression from M. tuberculosis infection to tuberculosis disease. PLoS Pathog.

[5] Lüscher B (2021). ADP-ribosyltransferases, an update on function and nomenclature. FEBS J.

[6] Juszczynski P (2006). BAL1 and BBAP are regulated by a gamma interferon-responsive bidirectional promoter and are overexpressed in diffuse large B-cell lymphomas with a prominent inflammatory infiltrate. Mol Cell Biol.

[7] Iwata H (2016). PARP9 and PARP14 cross-regulate macrophage activation via STAT1 ADP-ribosylation. Nat Commun.

[8] Yan Q (2013). BAL1 and its partner E3 ligase, BBAP, link poly(ADP-ribose) activation, ubiquitylation, and double-strand DNA repair independent of ATM, MDC1, and RNF8. Mol Cell Biol.

[9] Zhang Y (2015). PARP9-DTX3L ubiquitin ligase targets host histone H2BJ and viral 3C protease to enhance interferon signaling and control viral infection. Nat Immunol.

[10] Xing J (2021). Identification of poly(ADP-ribose) polymerase 9 (PARP9) as a noncanonical sensor for RNA virus in dendritic cells. Nat Commun.

[11] Chen Y-C (2020). Whole genome DNA methylation analysis of active pulmonary tuberculosis disease identifies novel epigenotypes: PARP9/miR-505/RASGRP4/GNG12 gene methylation and clinical phenotypes. Int J Mol Sci.

[12] Thompson EG (2018). Prospective discrimination of controllers from progressors early after low-dose Mycobacterium tuberculosis infection of cynomolgus macaques using blood RNA signatures. J Infect Dis.

[13] Zak DE (2016). A blood RNA signature for tuberculosis disease risk: a prospective cohort study. Lancet.

[14] https://www.proteinatlas.org/ENSG00000138496-PARP9/tissue.

[15] https://www.proteinatlas.org/ENSG00000138496-PARP9/celltype.

[16] Russo LC (2021). The SARS-CoV-2 Nsp3 macrodomain reverses PARP9/DTX3L-dependent ADP-ribosylation induced by interferon signaling. J Biol Chem.

[18] Ishikawa H, Barber GN (2008). STING is an endoplasmic reticulum adaptor that facilitates innate immune signalling. Nature.

[19] Ishikawa H (2009). STING regulates intracellular DNA-mediated, type I interferon-dependent innate immunity. Nature.

[20] Liao D (2013). The role of superoxide dismutase in the survival of Mycobacterium tuberculosis in macrophages. Jpn J Infect Dis.

[21] Anders S, Huber W (2010). Differential expression analysis for sequence count data. Genome Biol.

[22] Benjamini Y, Hochberg Y (1995). Controlling the false discovery rate: a practical and powerful approach to multiple testing. J R Stat Soc Series B Stat Methodol.

[23] Göbel K (2018). The coagulation factors fibrinogen, thrombin, and factor XII in inflammatory disorders-a systematic review. Front Immunol.

[24] Noris M, Remuzzi G (2013). Overview of complement activation and regulation. Semin Nephrol.

[25] Barrington RA (2009). Uncoupling CD21 and CD19 of the B-cell coreceptor. Proc Natl Acad Sci U S A.

[26] Sim RB, Laich A (2000). Serine proteases of the complement system. Biochem Soc Trans.

[27] The Gene Ontology Consortium (2021). The Gene Ontology resource: enriching a GOld mine. Nucleic Acids Res.

[28] Howe KL (2021). Ensembl 2021. Nucleic Acids Res.

[29] Yang CS (2017). Ubiquitin modification by the E3 ligase/ADP-ribosyltransferase Dtx3L/Parp9. Mol Cell.

[30] Penn-Nicholson A (2019). Discovery and validation of a prognostic proteomic signature for tuberculosis progression: a prospective cohort study. PLoS Med.

[31] Howard NC (2018). Mycobacterium tuberculosis carrying a rifampicin drug resistance mutation reprograms macrophage metabolism through cell wall lipid changes. Nat Microbiol.

[32] Ji J (2017). Phosphorylated fraction of H2AX as a measurement for DNA damage in cancer cells and potential applications of a novel assay. PLoS One.

[33] Podhorecka M (2010). H2AX phosphorylation: its role in DNA damage response and cancer therapy. J Nucleic Acids.

[34] Moreira-Teixeira L (2020). Mouse transcriptome reveals potential signatures of protection and pathogenesis in human tuberculosis. Nat Immunol.

[35] Mayer-Barber Katrin D (2011). Innate and adaptive interferons suppress IL-1α and IL-1β production by distinct pulmonary myeloid subsets during Mycobacterium tuberculosis infection. Immunity.

[36] Mayer-Barber KD, Yan B (2017). Clash of the cytokine titans: counter-regulation of interleukin-1 and type I interferon-mediated inflammatory responses. Cell Mol Immunol.

[37] McNab FW (2014). Type I IFN induces IL-10 production in an IL-27-independent manner and blocks responsiveness to IFN-γ for production of IL-12 and bacterial killing in Mycobacterium tuberculosis-infected macrophages. J Immunol.

[38] Mourik BC (2017). Interactions between type 1 interferons and the Th17 response in tuberculosis: lessons learned from autoimmune diseases. Front Immunol.

[39] Lochab S (2020). Mycobacterium tuberculosis exploits host ATM kinase for survival advantage through SecA2 secretome. Elife.

[40] Pilger D (2021). Interfaces between cellular responses to DNA damage and cancer immunotherapy. Genes Dev.

[41] Gonzalez-Hunt CP (2018). DNA damage by oxidative stress: measurement strategies for two genomes. Curr Opin Toxicol.

[42] Carpanini SM (2019). Therapeutic inhibition of the complement system in diseases of the central nervous system. Front Immunol.

[43] Jodele S (2020). Complement blockade for TA-TMA: lessons learned from a large pediatric cohort treated with eculizumab. Blood.

[44] Roy ER (2020). Type I interferon response drives neuroinflammation and synapse loss in Alzheimer disease. J Clin Invest.

[45] Nesargikar PN (2012). The complement system: history, pathways, cascade and inhibitors. Eur J Microbiol Immunol (Bp).

[46] Guo RF, Ward PA (2005). Role of C5a in inflammatory responses. Annu Rev Immunol.

[47] Robert I (2017). Robust immunoglobulin class switch recombination and end joining in Parp9-deficient mice. Eur J Immunol.

[48] Khader SA (2007). IL-23 and IL-17 in the establishment of protective pulmonary CD4+ T cell responses after vaccination and during Mycobacterium tuberculosis challenge. Nat Immunol.

[49] Braciale TJ (1977). Immunologic recognition of influenza virus-infected cells. II. Expression of influenza A matrix protein on the infected cell surface and its role in recognition by cross-reactive cytotoxic T cells. J Exp Med.

[50] Treerat P (2017). Novel role for IL-22 in protection during chronic Mycobacterium tuberculosis HN878 infection. Mucosal Immunol.

[51] Dejana E (1982). Bleeding time in rats: a comparison of different experimental conditions. Thromb Haemost.

[52] Guarini S (1996). A highly reproducible model of arterial thrombosis in rats. J Pharmacol Toxicol Methods.

[53] Gopal R (2013). S100A8/A9 proteins mediate neutrophilic inflammation and lung pathology during tuberculosis. Am J Respir Crit Care Med.

[54] Bolger AM (2014). Trimmomatic: a flexible trimmer for Illumina sequence data. Bioinformatics.

[55] Yates AD (2020). Ensembl 2020. Nucleic Acids Res.

[56] Dobin A (2013). STAR: ultrafast universal RNA-seq aligner. Bioinformatics.

[57] Leinonen R (2011). The sequence read archive. Nucleic Acids Res.

[58] Liao Y (2014). featureCounts: an efficient general purpose program for assigning sequence reads to genomic features. Bioinformatics.

[59] Fabregat A (2018). The reactome pathway knowledgebase. Nucleic Acids Res.

[60] Wang J (2017). WebGestalt 2017: a more comprehensive, powerful, flexible and interactive gene set enrichment analysis toolkit. Nucleic Acids Res.

[61] Shannon P (2003). Cytoscape: a software environment for integrated models of biomolecular interaction networks. Genome Res.

