# Supplementary material for: Poly(ADP-ribose) polymerase 9 mediates early protection against Mycobacterium tuberculosis infection by regulating type I IFN production
Source: J Clin Invest. 2023 Jun 15;133(12):e158630. doi: 10.1172/JCI158630 (PMC10266794; doi:10.1172/JCI158630)
Supplement: Supplemental data [file jci-133-158630-s290.pdf]

Supplementary Data

Table S1: Complete sample metadata and accession information for *Mtb* HN878-infected B6 and *Parp9*$^{-/-}$ mouse RNA sequencing study. (RNAseq data were deposited at the Sequence Read Archive (SRA) on the NCBI website https://www.ncbi.nlm.nih.gov/sra under BioProject accession number PRJNA753056.)

Table S2: Read counts, relative gene expression levels, gene annotations, and differential expression data (four comparisons) for every mouse gene available by RNA sequencing from B6 and *Parp9*$^{-/-}$ mice (RNAseq data were deposited at the Sequence Read Archive (SRA) on the NCBI website https://www.ncbi.nlm.nih.gov/sra under BioProject accession number PRJNA753056.)

Supplementary Figure1.

Gene	Mouse					Human	
	CFU:expression correlation		Significant differential gene expression direction			Significant spline on days to TB plot	
	Direction	P value	Controller / Naive	Progressor / Naive	Progressor / Controller	Direction	P value
PARP1	Up	0.0033	Up	Up	-	-	-
PARP2	Down	0.013	-	-	-	-	-
PARP3	-	-	-	-	Up	-	-
PARP4	Up	0.018	Up	Up	-	-	-
PARP5	-	-	Up	Up	-	Down	0.044
PARP6	Down	1.2E-07	-	-	Down	-	-
PARP7	-	-	-	Up	-	-	-
PARP8	Up	$<10^{-10}$	Up	Up	Up	-	-
**PARP9**	Up	$<10^{-10}$	Up	Up	Up	Up	0.0011
**PARP10**	Up	1.1E-08	Up	Up	Up	Up	0.015
PARP11	Up	$<10^{-10}$	Up	Up	Up	-	-
PARP12	Up	$<10^{-10}$	Up	Up	Up	-	-
PARP13	Up	$<10^{-10}$	Up	Up	Up	-	-
**PARP14**	Up	$<10^{-10}$	Up	Up	Up	Up	0.0014
PARP15	(No mouse ortholog)		(No mouse ortholog)			-	-
**PARP16**	Down	2.8E-08	Down	Down	-	Down	0.0027

Supplementary Figure 1: Bacterial CFU: expression correlation pattern for the PARP isoforms

Genetically diverse outbred (DO) or *Parp9*$^{-/-}$ mice were infected with *Mtb* HN878 (100 CFU) by the aerosol route. RNA isolated from mouse lung homogenates, collected at 30 dpi, was subjected to bulk RNA sequencing. The Pearson correlations and associated *P* values (based on the number of samples) between the detected bacterial load in the lungs (CFU) and the gene expression level of PARP genes are shown in the first column. Significant differential expression patterns with human TB are also shown for all the differentially expressed PARP isoforms (based on Ahmed et al. 2020(1)), as well as significant associations with TB progression in humans (based on Scriba et al. 2017(2)). "Up" refers to upregulation of PARP genes in association with TB, and "Down" refers to downregulation of PARP genes in association with TB.

Supplementary Figure 2.

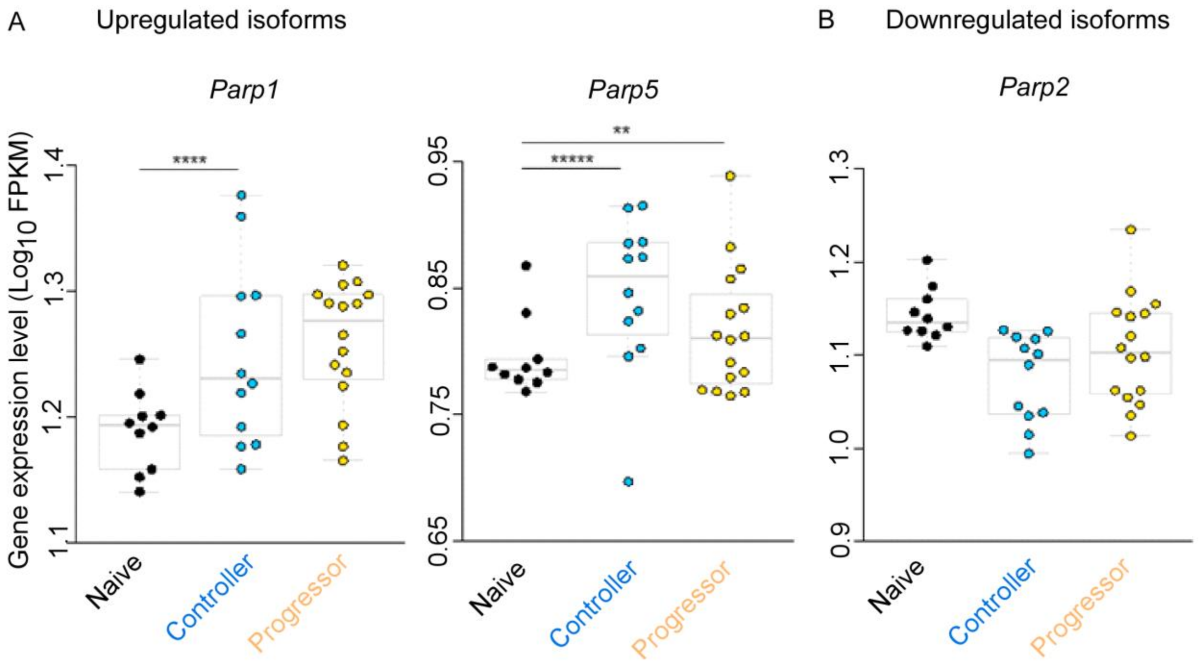

Supplementary Figure 2: Expression of parylating ADP ribosylated PARP isoforms in DO mice progressors

Genetically diverse outbred (DO) mice were infected with *Mtb* HN878 (100 CFU) by the aerosol route. RNA was isolated from mouse lung homogenates, collected at 30 dpi, and subjected to bulk RNA sequencing. Expression of poly ADP ribosylated PARP isoforms obtained from controllers and progressor *Mtb*-infected DO mice. (A) Upregulated isoforms and (B) downregulated isoforms. All *P*-values shown on the expression swarm plots represent FDR-corrected significance values for differential expression calculated by DESeq2 (naïve n = 10, controller n = 12 and progressor n = 16). ***** $P < 10^{-5}$, **** $P < 10^{-4}$, **$P<10^{-2}$. Data is derived from datasets from Ahmed et al. 2020 (1).

Supplementary Figure 3.

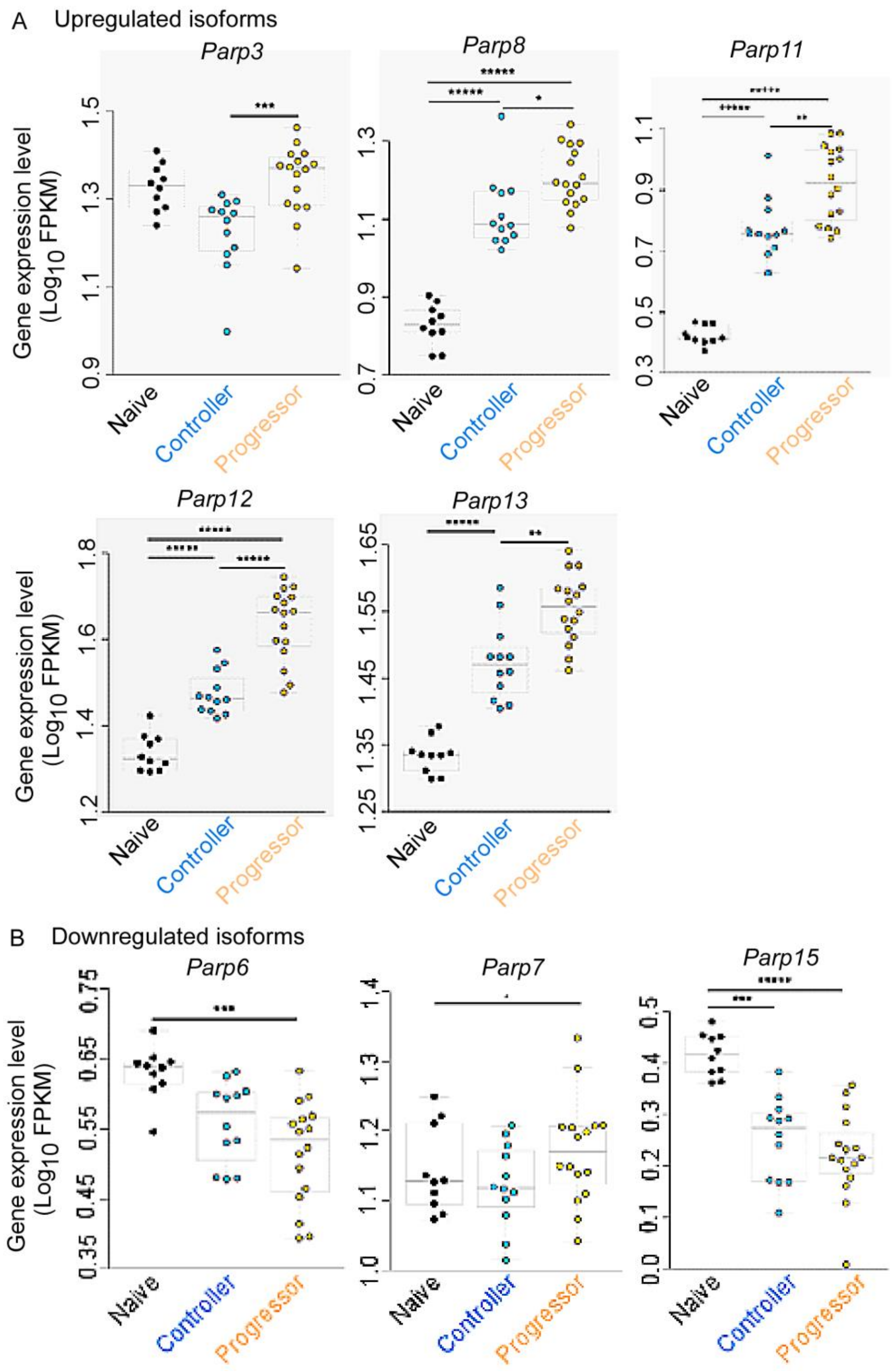

Supplementary Figure 3: Expression of mono ADP ribosylated PARP isoforms in DO mice

Genetically diverse outbred (DO) were infected with *Mtb* HN878 (100 CFU) by the aerosol route. RNA isolated from mouse lung homogenates, collected at 30 dpi was subjected to bulk RNA sequencing. Expression of upregulated (A) and downregulated (B) mono ADP ribosylated PARP isoforms across the lung transcriptional profiles obtained from controllers and progressor *Mtb*-infected DO mice. All *P*-values shown on the expression swarm plots represent FDR-corrected significance values for differential expression calculated by DESeq2 (naïve n = 10, controller n = 12 and progressor n = 16). ***** $P < 10^{-5}$, *** $P < 10^{-3}$, **$P<10^{-2}$. Data is derived from datasets from Ahmed et al. 2020(1).

Supplementary Figure 4:

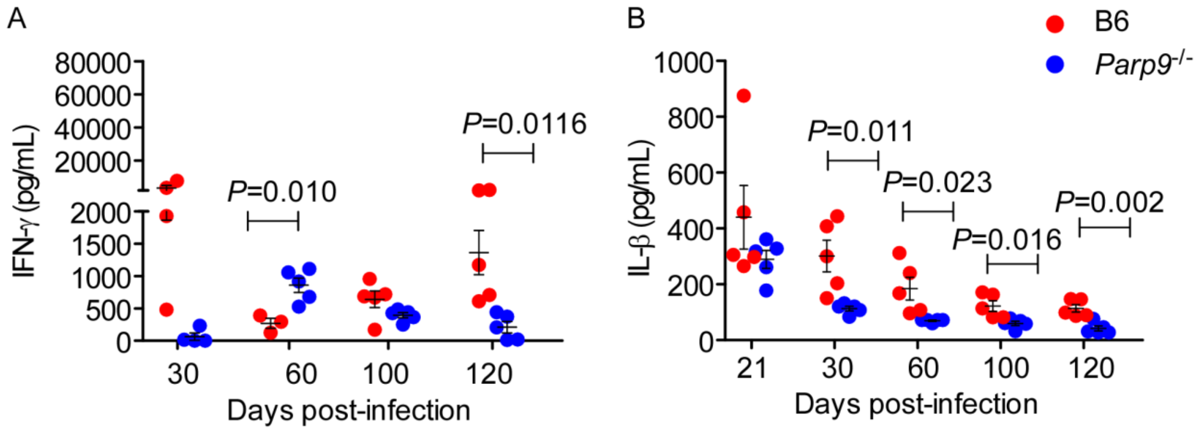

Supplementary Figure 4: IFN-γ and IL-1βproduction is decreased in *Mtb*-infected *Parp9*$^{-/-}$ mice

B6 or *Parp9*$^{-/-}$ mice were infected with *Mtb* HN878 (100 CFU) by the aerosol route. (A) IFN-γ and (B) IL-1β were measured in lung homogenates on 30, 60, 100 and 120 dpi by ELISA. The data points represent the mean ± SEM, n = 5 per group. Significance was determined by unpaired two-tailed students t-test. $P \leq 0.05$ is considered significant.

Supplementary Figure 5

A

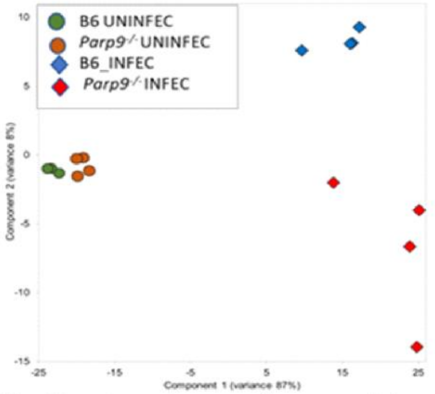

B

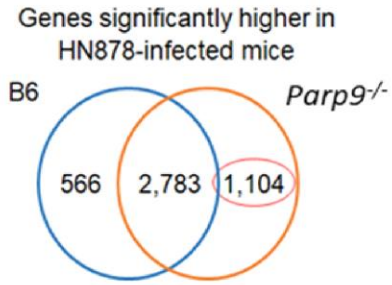

C Significant reactome pathway enrichment among the 1,104 genes significantly higher during *Mtb* HN878 infection in *Parp9*-/- but not B6 mice

Reactome pathway	Description	Total pathway size	# Significant genes	FDR-corrected P value
R-MMU-1430728	Metabolism	1810	212	0
R-MMU-71291	Metabolism of amino acids and derivatives	250	48	3.5E-08
R-MMU-140877	Formation of Fibrin Clot (Clotting Cascade)	39	17	8.7E-08
R-MMU-140837	Intrinsic Pathway of Fibrin Clot Formation	23	12	2.6E-06
R-MMU-174824	Plasma lipoprotein assembly, remodeling, and clearance	62	19	5.0E-06
R-MMU-8852276	The role of GTSE1 in G2/M progression after G2 checkpoint	70	20	6.6E-06
R-MMU-166658	Complement cascade	48	16	1.3E-05
R-MMU-382556	ABC-family proteins mediated transport	97	23	2.1E-05
R-MMU-211859	Biological oxidations	251	41	2.5E-05
R-MMU-156588	Glucuronidation	24	11	2.5E-05
R-MMU-8963899	Plasma lipoprotein remodeling	25	11	3.8E-05
R-MMU-5358346	Hedgehog ligand biogenesis	60	17	4.1E-05
R-MMU-977606	Regulation of Complement cascade	42	14	4.1E-05
R-MMU-69229	Ubiquitin-dependent degradation of Cyclin D1	48	15	4.1E-05
R-MMU-75815	Ubiquitin-dependent degradation of Cyclin D	48	15	4.1E-05
R-MMU-351202	Metabolism of polyamines	82	20	4.3E-05
R-MMU-69601	Ubiquitin Mediated Degradation of Phosphorylated Cdc25A	49	15	4.3E-05
R-MMU-69610	p53-Independent DNA Damage Response	49	15	4.3E-05
R-MMU-69613	p53-Independent G1/S DNA damage checkpoint	49	15	4.3E-05
R-MMU-5607761	Dectin-1 mediated noncanonical NF-kB signaling	56	16	4.7E-05

D Formation of fibrin clot (clotting cascade) genes significantly higher *Mtb* HN878 infection in *Parp9*-/- mice but not B6 mice

Gene ID	Name	Description
ENSMUSG00000028001	Fga	fibrinogen alpha chain
ENSMUSG00000033860	Fgg	fibrinogen gamma chain
ENSMUSG00000033831	Fgb	fibrinogen beta chain
ENSMUSG00000027249	F2	coagulation factor II
ENSMUSG00000022875	Kng1	kininogen 1
ENSMUSG00000026715	Serpinc1	serine (or cysteine) peptidase inhibitor, clade C (antithrombin), r
ENSMUSG00000021492	F12	coagulation factor XII (Hageman factor)
ENSMUSG00000047953	Gp5	glycoprotein 5 (platelet)
ENSMUSG00000024386	Proc	protein C
ENSMUSG00000060459	Kng2	kininogen 2
ENSMUSG00000031138	F9	coagulation factor IX
ENSMUSG00000050675	Gp1ba	glycoprotein 1b, alpha polypeptide
ENSMUSG00000031443	F7	coagulation factor VII
ENSMUSG00000029373	Pf4	platelet factor 4
ENSMUSG00000022766	Serpind1	serine (or cysteine) peptidase inhibitor, clade D, member 1
ENSMUSG00000109764	Klkb1	kallikrein B, plasma 1
ENSMUSG00000031645	F11	coagulation factor XI

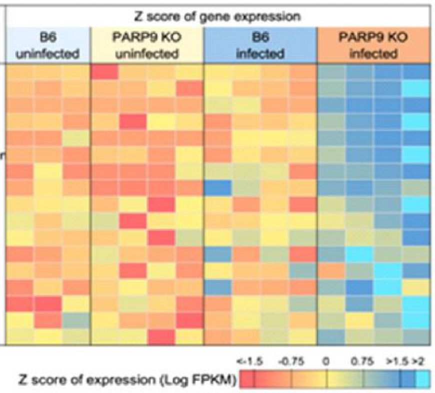

Supplementary Figure 5. Fibrin clotting cascade genes are upregulated in *Mtb*-infected *Parp9*-/- mice.

B6 or *Parp9*$^{-/-}$ mice were infected with *Mtb* HN878 (100 CFU) by the aerosol route and RNA isolated from mouse lung homogenates (30dpi) was subjected to bulk RNA sequencing. (A) Principal component analysis (PCA) plot of the mouse RNA-seq samples showing that most of the variance between samples (87%) comes from differences between uninfected vs. infected mice, but *Parp9*$^{-/-}$ has distinct expression profiles from B6, especially after infection. (B) Venn diagram showing overlap of genes significantly higher in *Mtb* HN878-infected B6 and *Parp9*$^{-/-}$ mice. (C) Significant Reactome pathway enrichment among the 1,104 genes considerably higher during *Mtb* HN878 infection in *Parp9*$^{-/-}$ but not B6 mice and (D) heat map showing upregulation of clotting cascade genes during *Mtb* HN878 infection in *Parp9*$^{-/-}$ mice but not B6 mice.

Supplementary Figure 6.

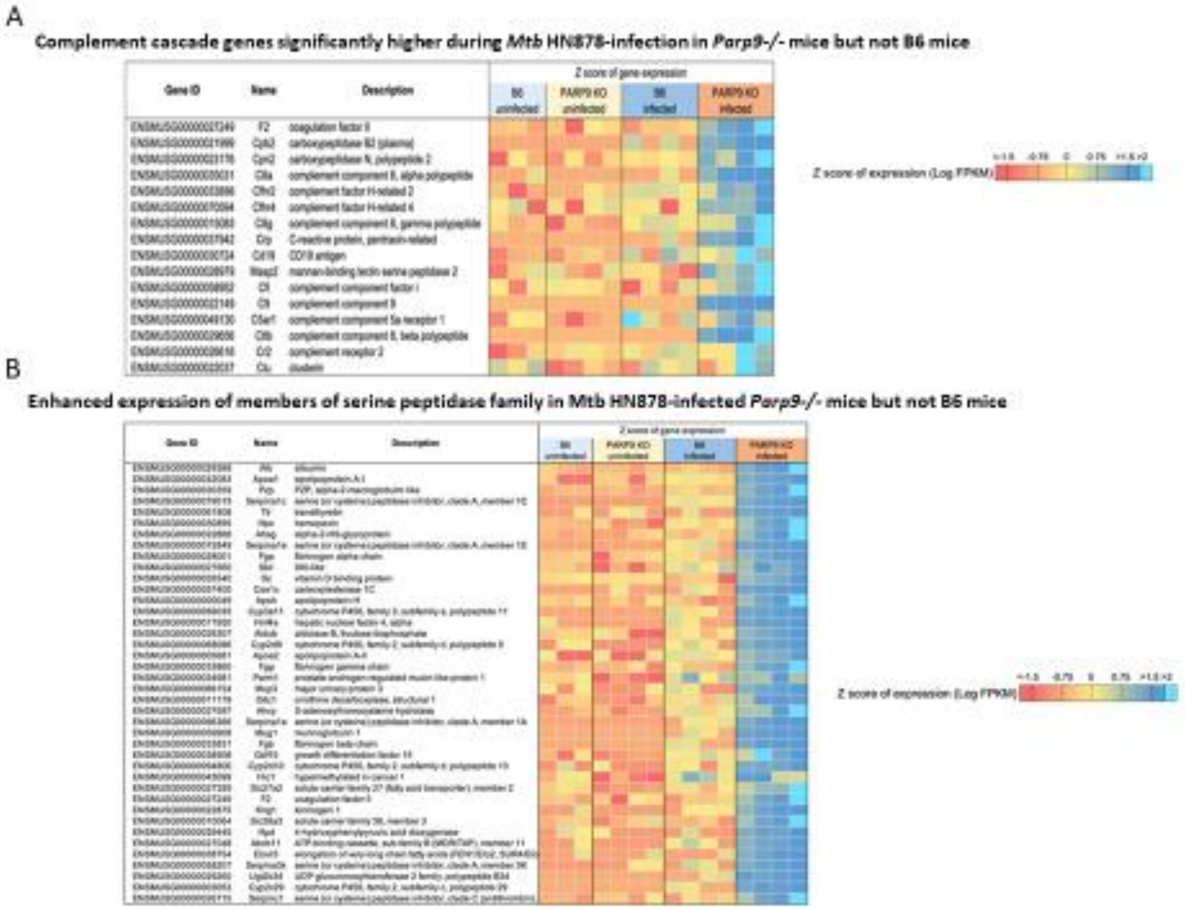

Supplementary Figure 6. Genes belonging to the complement cascade and the serine peptidase family are upregulated in *Mtb* HN878-infected *Parp9*$^{-/-}$ mice

B6 or *Parp9-/-* mice were infected with *Mtb* HN878 (100 CFU) by the aerosol route and RNA derived from lung homogenates of mice at 30dpi was subjected to bulk RNA sequencing. (A) heat map showing upregulation of complement cascade genes during *Mtb* HN878 infection in *Parp9*$^{-/-}$ mice but not B6 mice. (B) heat map showing upregulation of serine-peptidase family members during *Mtb* HN878-infection in *Parp9*$^{-/-}$ mice but not B6 mice. Significantly differentially expressed genes between B6 and *Parp9*$^{-/-}$ *Mtb*-

infected lungs were identified using DESeq2 P value significance threshold of 0.05 (after FDR correction).

Supplementary Figure 7.

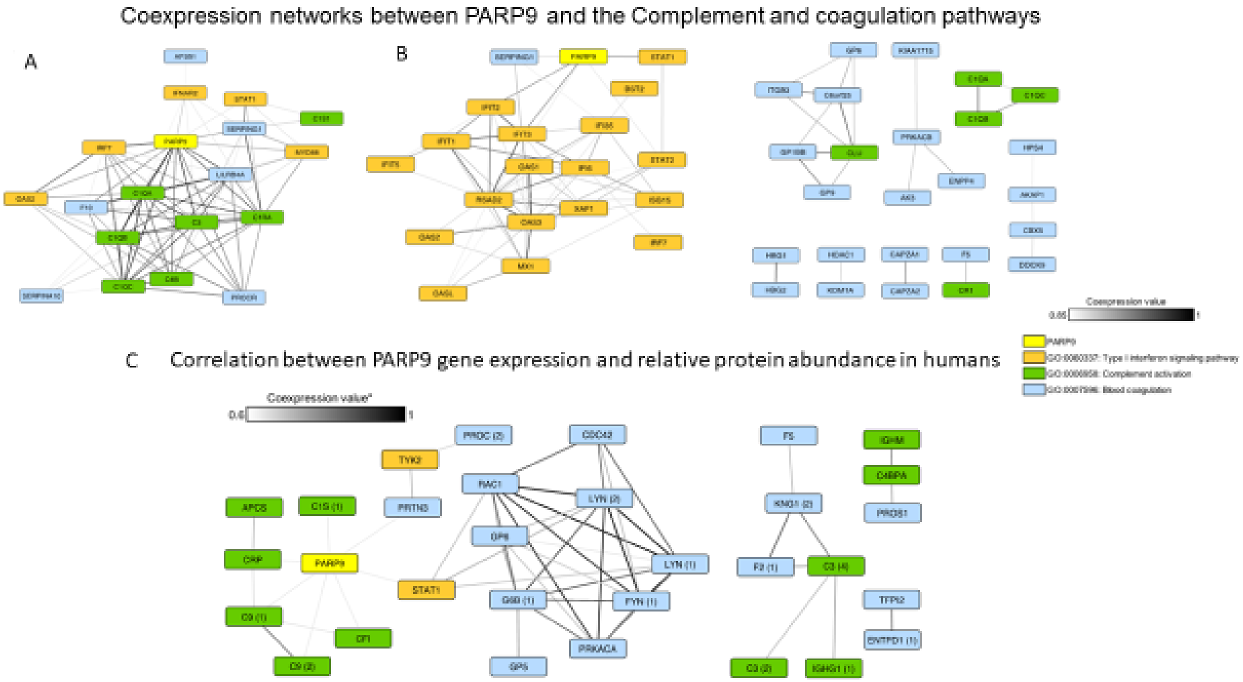

Supplementary Figure 7. Coexpression and correlation between Parp9 and members of the complement and coagulation pathways

Gene co-expression networks based on all RNA-seq data analyzed in the (A) DO mouse immune correlates and (B) human adolescent cohort study (ACS) (3)and the mouse DO immune correlates (1). Within each species' dataset, Pearson correlation values between pairs of two genes were calculated, quantifying the similarity of their expression (normalized gene expression data; Log FPKM) across all samples analyzed. (C) Correlation between *PARP9* gene expression and relative protein abundance in humans,

using a previously-published protein microarray by using Pearson correlation values (4) (Penn-Nicholson 2019).

References

1. Ahmed M, Thirunavukkarasu S, Rosa BA, Thomas KA, Das S, Rangel-Moreno J, et al. Immune correlates of tuberculosis disease and risk translate across species. *Science Translational Medicine.* 2020;12(528):eaay0233.
2. Scriba TJ, Penn-Nicholson A, Shankar S, Hraha T, Thompson EG, Sterling D, et al. Sequential inflammatory processes define human progression from M. tuberculosis infection to tuberculosis disease. *PLoS Pathog.* 2017;13(11):e1006687-e.
3. Zak DE, Penn-Nicholson A, Scriba TJ, Thompson E, Suliman S, Amon LM, et al. A blood RNA signature for tuberculosis disease risk: a prospective cohort study. *The Lancet.* 2016;387(10035):2312-22.
4. Penn-Nicholson A, Hraha T, Thompson EG, Sterling D, Mbandi SK, Wall KM, et al. Discovery and validation of a prognostic proteomic signature for tuberculosis progression: A prospective cohort study. *PLOS Medicine.* 2019;16(4):e1002781.
